# Evaluating the Impact of Drug Regulation Stringency on Global Drug-Use Patterns: A Cross-Sectional Study

**DOI:** 10.1016/j.focus.2026.100499

**Published:** 2026-04-01

**Authors:** Konstantine Chakhunashvili, Giorgi Meladze, Eka Kvirkvelia, Davit G. Chakhunashvili

**Affiliations:** a Caucasus Medical School, Caucasus University, Tbilisi, Georgia; b Reproductive Education Hub, Tbilisi, Georgia; c School of Law, Ilia State University, Tbilisi, Georgia; d Department of Gynecology, Caucasus University, Tbilisi, Georgia; e Department of Pediatrics, Alte University, Tbilisi, Georgia; f Freie Universitat Berlin, Berlin, Germany

**Keywords:** Addiction, drug abuse, regulatory framework

## Abstract

**Introduction:**

Global drug policies vary widely in their level of restrictiveness and alignment with human rights and health standards. However, the relationship between national drug policy frameworks and drug-use prevalence remains poorly understood. This study aimed to evaluate whether the stringency or rights-based orientation of national drug policies is associated with the prevalence of injection drug use and illicit drug use across countries.

**Methods:**

A cross-sectional ecologic analysis was conducted using publicly available data from international databases. An overall drug policy score was developed to quantify policy stringency, and the Global Drug Policy Index was included to assess rights-based policy quality. Additional variables included Democracy Index, gross domestic product per capita, alcohol consumption, and Corruption Perceptions Index. Multivariate linear regression was used to identify predictors of injection drug-use and illicit drug-use prevalence.

**Results:**

Whereas bivariate analysis revealed several significant correlations of policy, socioeconomic, and behavioral indicators with drug-use prevalence, multivariate analysis showed that neither overall drug policy score nor Global Drug Policy Index significantly predicted injection drug use or illicit drug use. For injection drug use, only alcohol consumption (*p*=0.006) and Democracy Index (*p*=0.016) remained significant in the model, which explained 61% of the variance (R²=0.61, *p*=0.015). Only alcohol consumption (*p*=0.032) emerged for illicit drug use in the full model despite a good overall fit (R²=0.61, *p*=0.001).

**Conclusions:**

Because this study uses a cross-sectional ecologic design, the findings identify associations at the country level but cannot establish causality. The association between regulation strictness and drug-use prevalence was not detected. The observed relationships—such as higher reported drug use in less corrupt or more rights-oriented countries—may reflect differences in data quality or reporting practices rather than true differences in prevalence. The findings might indicate the need to adopt more comprehensive approach in regard to drug-use prevalence rather than a legal framework, underlining the need for incorporating social, cultural, and behavioral factors in understanding drug-use patterns.

## Highlights

• Global analysis of drug policy stringency versus drug-use prevalence was performed.
• Policy scores are not predictive; governance and socioeconomic factors are stronger.
• Alcohol use is consistently linked to drug use at population level.

## Graphical abstract

**Figure:**
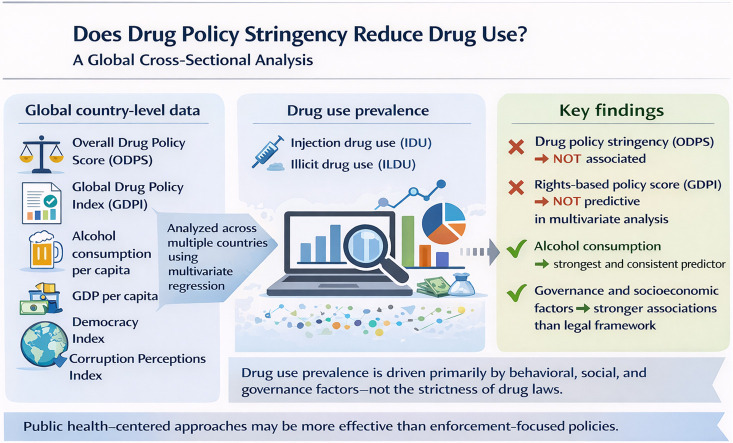

## INTRODUCTION

Substance use remains a significant health issue across both adult and pediatric populations, showing a 23% increase over the past decade despite stringent regulations enforced by the majority of the world’s countries.^1^, ^2^, ^3^ The most recent estimates place the global illicit drug trade market size between 320 billion and 500 billion U.S. Dollars annually.^2^ The black market for drugs is expanding rapidly, leveraging electronic commerce through the dark web.^4^ Despite efforts by various drug enforcement agencies, such as shutting down Silk Road by the Federal Bureau of Investigation (involving over 100,000 customers) and DarkMarket in Germany (with 2,400 drug sellers), these markets quickly adapt by migrating to other platforms.^4^^,^^5^ Drug policy is often grounded in the principles of deterrence theory, which assumes that harsher penalties and stricter enforcement reduce substance use by increasing the perceived costs of engaging in such behavior. Within this model, criminalization and punitive sanctions are expected to discourage initiation and limit prevalence. Yet, evidence increasingly demonstrates that stringent regulation may instead displace markets underground, foster organized crime, and exacerbate health harms, raising questions about the adequacy of deterrence as a sole explanatory framework for drug-use patterns. The public health and human rights paradigm emphasize that drug use is not solely a matter of law enforcement but also a social and medical issue. Policies aligned with harm reduction, access to treatment, and the protection of human rights—captured in composite measures such as the Global Drug Policy Index (GDPI)—are theorized to mitigate the negative consequences of drug use even if they do not directly reduce prevalence. This perspective situates substance use within a broader governance framework, where legitimacy derives not only from control but also from safeguarding health and rights. Beyond regulatory frameworks, socioeconomic and cultural determinants play an equally critical role. Economic development, measured by gross domestic product (GDP) per capita, may influence access and affordability of substances, whereas governance quality, reflected in the Corruption Perceptions Index (CPI) and Democracy Index (DI), shapes enforcement, data accuracy, and civic attitudes toward drugs. At the cultural level, alcohol consumption serves as a proxy for general substance-use norms, consistent with the gateway hypothesis,^6^ whereby permissive behaviors toward one substance may increase likelihood of others. Although modern debates on regulating substances listed under Schedules I and II of the 1961 Single Convention on Narcotic Drugs have persisted for just over a century, many of these substances have been used since immemorial time.^7^^,^^8^ Understanding why some substances attract regulatory attention requires considering cultural, religious, sociopolitical, and economic factors.^9^ The review does not aim to cover this history exhaustively but to outline key themes relevant to central objective: assessing global data to examine how regulatory strength—quantified by research team—relates to drug-use prevalence.

Historical evolution of drug regulation reveals several paradoxes. The same substances have been treated as medicine, habit, crime, or illness at different times. Marijuana, for example, now illegal in many countries, was once considered nonaddictive and medicinal.^10^ Early public debates often lumped together commodities now freely traded with those regulated or restricted; some advocates even sought limits on tea and coffee alongside tobacco and alcohol.^11^ Braithwaite and Drahos describe 5 major strands of regulatory development: global illicit-drug regulation, expanding prescription-drug regulation, pharmaceutical production and marketing oversight, national regulation of alcohol and tobacco, and food regulation.^12^

Drug regulation emerged unevenly across jurisdictions^13^^,^^14^ but became global in the early 20th century.^15^ Whereas many link international frameworks to U.S. influence,^16^ others highlight earlier Asian regulatory experiences.^17^ Historical analyses suggest distinct national and international trajectories,^18^ with key milestones including the 1909 Shanghai Opium Commission and The 1912 Hague International Opium Convention.^19^ Technological change—particularly the internet—now challenges enforcement and exposes persistent regulatory gaps.^20^^,^^21^ Questions are also raised about reconciling national decriminalization with international obligations.^22^

Policy effects are mixed. In the U.S., marijuana legalization has reduced some crime but also fueled cross-state black-market activity where it remains illegal.^23^ Drug prohibition more broadly has driven organized crime and violence because criminal organizations exploit lucrative markets, and enforcement efforts destabilize power dynamics.^24^^,^^25^ Similar patterns appeared during alcohol prohibition, when violence brokers amplified violent networks in Chicago.^26^

Financial costs of enforcement are substantial: Australia spends about $700 million annually, the U.S. spends an estimated $47 billion, and global spending exceeds $100 billion.^27^, ^28^, ^29^ Yet, drug use persists; in Australia, nearly 1 in 10 people aged >14 years use illicit drugs yearly, and 40% have tried them.^29^ Criminal penalties also fail to deter use,^30^ and the data analysis supports this. Conviction and imprisonment negatively affect long-term employment and earnings.^31^ High drug prices drive dependent users toward crime,^32^ suggesting that lower prices could reduce related offenses.^33^ As with drug prohibition, violence around gambling in the U.S. diminished markedly once legalized.^34^

Prohibition can also foster corruption, undermine trust in institutions,^35^ and exacerbate health harms. Fear of arrest may lead heroin users to share needles or inject quickly, increasing HIV and hepatitis C transmission.^36^ Overdose, infection, and lost productivity further reflect the public-health burden.^37^, ^38^, ^39^ Harm-reduction strategies—including in prisons—are essential.^40^

The U.S. opioid crisis illustrates the urgency for health-centered approaches; despite debate, expanding naloxone access is widely supported.^41^^,^^42^ With overdose incidents rising and an economic burden approaching $500 billion, rapid action is required.^41^, ^42^, ^43^ Portugal’s health-centered reforms led to more than 80% reductions in overdose deaths, dramatic declines in HIV transmission (52% to 6%), and more than 40% drop in incarceration, largely owing to expanded treatment and harm-reduction services.^43^^,^^44^ Switzerland’s four-pillar policy—prevention, therapy, harm reduction, and repression—has similarly reduced harms, supported social reintegration, and cut intravenous (IV) drug-use prevalence by about 35% since the 1990s.^43^, ^44^, ^45^, ^46^, ^47^, ^48^ Together, these perspectives suggest an integrative model in which drug-use prevalence arises from the interplay between legal deterrence, rights-based policy orientation, socioeconomic governance, and cultural behavioral indicators. This framework provides the conceptual foundation for this analysis, which evaluates whether national drug policy stringency or rights-based orientation can predict drug-use prevalence when considered alongside social, economic, and behavioral factor. In this research, drug policy stringency scoring system was developed, and comprehensive data collection process was conducted to assign each country an overall drug policy score (ODPS). In addition, data on the estimated prevalence of injection and illicit drug use, alcohol consumption rates, GDP per capita, GDPI, DI, and the CPI were collected. The aim was to perform an in-depth analysis and identify potential correlations between these variables. The hypothesis was that stricter national drug regulation policies are not associated with lower prevalence of injection or illicit drug use; instead, factors such as alcohol consumption, GDP per capita, DI, and corruption perception have stronger predictive value for national drug-use patterns.

## METHODS

### Study Sample

The cross-sectional observational study was conducted between January and July 2024, focusing on determining the correlation between the strictness of local legislation, GDP per capita, corruption index, democracy, alcohol consumption, and the prevalence of IV drug use (IDU) and overall illicit drug use (ILDU) across multiple countries. The primary sources for quantified data (Table 1) included the World Bank,^49^ Economist Intelligence^50^ European Union Drug Agency,^51^ Harm Reduction International,^52^ Harm Reduction Consortium,^53^ Our World in Data and Institute of Health Metrics and Evaluation,^54^ Transparency International,^55^ Central Intelligence Agency,^56^ and other publications, including regional studies and official legislature of the respective countries (Appendix Material 1, available online).

**Table 1:** All the Quantified Data That Have Been Collected During the Research

Country/territory	Injecting drug-user prevalence per 100,000 population	Illicit drug-user prevalence per 100,000 population	GDP per capita (USD)	CPI	Strength of regulation, cannabis	Strength of regulation, hallucinogens and stimulants	Strength of regulation, IV drugs	Overall drug policy score	Pure alcohol per capita (L/year)
Afghanistan	120	400	352.6	20	5	4	4	13	0.01
Albania	350	600	8,367.8	37	4	3	4	11	4.40
Algeria		400	5260.2	36	5	4	4	13	0.59
American Samoa		700	19,673.4		4	4	4	12	
Andorra		900	46,544.7		3	3	4	10	10.99
Angola		300	2,309.5	33	4	4	4	12	5.84
Antigua and Barbuda		800	21,560		2	3	4	9	11.88
Argentina	40	800	13,730.5	37	1	2	3	6	7.95
Armenia	450	500	8,715.8	47	4	3	4	11	3.77
Australia	460	1,800	64,711.8		2	3	3	8	9.51
Austria		1,000	56,506	71	3	3	3	9	11.90
Azerbaijan	860	500	7,155.1	23	4	4	4	12	1.38
Bahrain		400	29,084.3	42	5	4	4	13	1.18
Bangladesh	30	300	2,529.1	24	4	4	4	12	0.00
Barbados		1,000	22,672.6	69	3	3	4	10	9.94
Belarus	1,250	700	7,829.1	37	4	4	4	12	10.57
Belgium	80	1,000	53,475.3	73	2	3	3	8	9.15
Belize		800	7,987.6		3	3	3	9	5.93
Benin		300	1,434.7	43	3	3	3	9	1.25
Bermuda		800	123,091.1		1	3	3	7	
Bhutan		300	3,704	68	4	4	4	12	0.07
Bosnia and Herzegovina	470	400	8,426.1	35	3	3	3	9	5.46
Botswana		400	7,249	59	4	4	4	12	5.98
Brazil	200	900	10,043.6	36	2	3	3	8	6.12
Brunei		800	33,430.9		5	4	4	13	0.69
Bulgaria	220	700	15,797.6	45	3	3	3	9	11.18
Burkina Faso		200	874.1	41	3	3	3	9	7.28
Burundi		300	199.6	20	3	3	3	9	4.07
Cambodia	40	500	1,875.1	22	2	3	3	8	4.56
Cameroon	20	300	1,673.6	27	3	3	3	9	4.09
Canada	370	2,300	53,371	76	0	1	1	2	8.00
Central African Republic	130	300	445	24	3	3	3	9	0.94
Chad	10	200	719.4	20	3	3	3	9	0.55
Chile	180	1,100	17,093.2	66	2	3	3	8	7.80
China	190	600	12,614.1	42	4	4	4	12	4.48
Colombia	100	800	6,979.7	40	2	3	3	8	4.09
Comoros		300	1,587.2	20	4	3	3	10	0.18
Congo	10	300	2,508.8	22	3	3	3	9	5.74
Democratic Republic of the Congo	360	300	649.1	20	4	3	3	10	0.56
Cook Islands		700			2	2	2	6	12.97
Costa Rica		500	16,595.4	55	2	3	3	8	3.07
Croatia	220	800	21,459.8	50	2	3	3	8	9.64
Czechia	600		30,427.4	41	3	2	2	7	12.73
Cuba		800	9,499.6	42	2	3	3	8	4.70
Cyprus	100	600	34,701.4	53	5	4	4	13	9.59
Denmark	360	1,100	67,967.4	90	4	3	4	11	9.16
Djibouti		300	3,606.4	30	3	3	3	9	0.21
Dominica		1,000	8,953.9	56	2	2	2	6	6.32
Dominican Republic	20	600	10,716	35	2	4	4	10	5.56
East Timor	10	500	1,648.6	43	4	3	3	10	
Ecuador		500	6,533.6	34	2	2	2	6	
Egypt	370	400	3,512.6	35	4	4	4	12	0.14
El Salvador	170	400	5,344.2	31	5	4	4	13	2.94
Equatorial Guinea		300	7,066	17	2	3	3	8	6.11
Eritrea		300	643.8	21	4	3	3	10	0.93
Estonia	1,000	1,900	29,823.7	76	3	3	3	9	11.65
Ethiopia		300	1,293.8	37	4	3	3	10	1.16
Fiji		700	5,868.2	52	2	3	3	8	2.71
Finland	740	1,200	53,755.9	87	5	4	4	13	8.23
France	270	1,100	44,460.8	71	5	4	4	13	11.44
Gabon		300	8,420.1	28	4	3	3	10	6.47
Georgia	224	500	8,120.4	53	2	3	3	8	7.45
Germany	500	1,000	52,745.8	78	3	3	3	9	10.56
Ghana		300	2,238.2	43	4	3	3	10	1.59
Greece	400	700	22,990	49	5	4	4	13	6.33
Greenland		1,700	57,116.3		5	4	4	13	
Grenada		900	10,463.6	53	5	4	4	13	8.62
Guam		700	40,227.3		0	4	4	8	1.63
Guatemala	30	400	5,797.5	23	4	4	4	12	
Guinea	30	200	1,663.9	26	4	4	4	12	0.33
Guinea-Bissau		200	914.3	22	5	4	4	13	3.21
Guyana		600	20,626.2	40	2	4	4	10	5.11
Haiti		700	1,693.1	17	4	4	4	12	2.85
Honduras		500	3,247.2	23	5	4	4	13	2.73
Hungary	100	700	13,138.3	42	5	4	4	13	10.79
Iceland	230	1,200	78,811.1	72	3	4	4	11	7.72
India	100	400	2,484.8	39	3	3	4	10	3.09
Indonesia	20	500	4,940.5	34	5	4	4	13	0.08
Iraq		400	5,512.5	23	5	4	4	13	0.16
Ireland	20	1,300	103,684.9	77	3	3	4	10	10.91
Israel	500	700	52,261.5	62	3	4	4	11	3.07
Italy	280	900	38,373.2	56	2	3	3	8	7.65
Jamaica		900	6,874.2	44	3	4	4	11	3.46
Japan	470	800	33,834.4	73	5	4	4	13	8.36
Jordan	20	400	4,482.1	46	5	4	4	13	0.25
Kazakhstan	700	800	13,136.6	39	5	3	4	12	3.73
Kenya	210	300	1,949.9	31	5	3	4	12	1.68
Kiribati		700	2,089.9		4	3	3	10	0.43
Kuwait	70	400	37,533.2	46	5	4	4	13	0.00
Kyrgyzstan	690	500	1,969.9	26	5	4	4	13	4.02
Laos	40	500	2,075.4	28	5	4	4	13	8.15
Latvia	610	900	23,184.3	60	5	4	4	13	12.90
Lebanon	80	500	3,350.3	24	5	4	4	13	1.14
Lesotho	210	400	878	39	5	4	4	13	3.56
Liberia	150	300	799.5	25	5	4	4	13	3.12
Libya	50	500	7330	18	5	4	4	13	0.01
Lithuania	460	1,000	27,102.8	34	3	4	4	11	11.93
Luxembourg	190	1,100	128,259.4	78	2	3	4	9	11.00
Madagascar	120	300	528.7	25	5	4	4	13	0.89
Malawi		300	672.8	15	5	4	4	13	
Malaysia	340	500	11,648.7	50	5	4	4	13	0.64
Maldives	240	600	12,667.4	39	5	4	4	13	1.38
Mali	80	200	897.4	28	5	4	4	13	0.60
Malta	310	900	37,882.3	51	2	3	4	9	8.07
Marshal Islands		700	6,762.5		5	4	4	13	0.00
Mauritania		200	2,149.4	30	5	4	4	13	
Mauritius	730	700	11,416.9	51	5	4	4	13	3.39
Mexico	130	500	13,926.1	31	2	4	4	10	4.25
Moldova	950	600	6,650	42	3	4	4	11	7.45
Mongolia		600	5,764.8	33	5	4	4	13	5.46
Montenegro	300	500	12,016.9	46	5	4	4	13	9.91
Morocco	10	400	3,672.1	38	5	4	4	13	0.51
Mozambique	30	300	608.4	25	5	4	4	13	1.46
Myanmar	260	500	1,187.6	20	5	4	4	13	2.06
Namibia		500	4,742.8	49	5	4	4	13	2.38
Nepal	200	300	1,324	35	5	4	4	13	0.36
Netherlands	100	1,000	62,536.2	79	2	4	4	10	8.23
New Zealand	610	1,900	48,527.8	85	3	4	4	11	9.17
Nicaragua		300	2,530.3	17	5	4	4	13	3.69
Niger		200	618.3	32	5	4	4	13	0.11
Nigeria	80	200	1,621.1	25	5	4	4	13	4.49
North Korea		600		17	5	4	4	13	3.61
Norway	220	1,100	87,961.8	84	5	4	4	13	6.05
Oman	90	400	23,295.3	43	5	4	4	13	0.47
Pakistan	400	400	1,407	29	5	4	4	13	0.04
Panama		500	18,661.8	35	5	4	4	13	6.54
Papua New Guinea		700	2,994.5	29	5	4	4	13	1.26
Paraguay	140	500	6,260.5	28	2	4	4	10	5.47
Peru		400	7,789.9	33	2	4	4	10	5.74
Philippines	70	500	3,725.6	34	5	4	4	13	4.85
Poland	10	800	22,112.9	54	3	3	3	9	10.96
Portugal	210	700	27,275.1	61	2	2	2	6	10.37
Puerto Rico	1,500	900	36,799.1		3	4	4	11	
Qatar		400	87,480.4	58	5	4	4	13	0.96
Romania	600	300	18,419.4	46	4	3	3	10	10.96
Russia	1,320	1,100	13,817.2	26	5	4	4	13	7.29
Rwanda	10	1,100	1,000.2	53	5	4	4	13	6.35
Saint Kitts and Nevis		800			3	4	4	11	8.84
Saint Lucia		900	13,980.1	55	2	4	4	10	9.30
Saint Vincent and the Grenadines		900	10,279.5	60	2	4	4	10	7.48
Samoa		900	4,139		5	4	4	13	2.18
Sao Tome and Principe		300	2,601.8	45	5	4	4	13	4.23
Saudi Arabia		400	28,895	52	5	4	4	13	0.00
Senegal	10	200	1,746	43	5	4	4	13	0.25
Serbia	340	500	1,1361	36	5	4	4	13	7.45
Seychelles	3,210	700	17,879.2	71	5	4	4	13	9.48
Sierra Leone	40	200	433.4	35	5	4	4	13	3.22
Singapore		800	8,4734.3	83	5	4	4	13	1.81
Slovakia	490	700	24,470.2	54	4	3	3	10	10.30
Slovenia	520	900	32,163.5	54	3	2	2	7	11.05
Solomon Islands		700	2,203.2	43	5	4	4	13	1.19
Somalia		300	643.8	11	5	4	4	13	0.00
South Africa	220	700	6,253.2	41	2	4	4	10	7.21
South Korea		800	33,121.4	63	5	4	4	13	7.74
South Sudan	280	300	1,071.8	13	5	4	4	13	
Spain	300	1,500	32,677	60	3	2	2	7	10.72
Sri Lanka	10	600	3,828	34	5	4	4	13	2.58
Sudan		400	2,272.5	20	5	4	4	13	1.93
Suriname		700	6,069	40	5	4	4	13	6.60
Sweden	130	1,000	56,305.3	82	5	4	4	13	7.10
Switzerland	750	1,300	99,994.9	82	3	2	2	7	9.41
Syria		300	421.1	13	5	4	4	13	0.13
Taiwan		700	32,679	67	5	4	4	13	
Tajikistan	410	500	1,189	20	5	4	4	13	
Tanzania	120	400	1,211.1	40	5	4	4	13	7.81
Thailand	150	800	7,171.8	35	2	4	4	10	6.86
Togo	60	200	1,013	31	5	4	4	13	1.40
Tonga		700	4,681.7		5	4	4	13	0.31
Trinidad and Tobago		700	18,333	42	2	4	4	10	5.81
Tunisia	130	400	3,895.4	40	5	4	4	13	1.51
Turkey	30	300	12,985.8	34	5	4	4	13	1.18
Turkmenistan		600	9,190.7	18	5	4	4	13	2.88
Tuvalu		700	5,265.1		5	4	4	13	0.93
Uganda	30	400	1,014.2	26	0	4	4	8	6.82
Ukraine	1,170	800	5,181.4	36	5	4	4	13	5.69
United Arab Emirates		600	52,976.8	68	5	4	4	13	2.03
United States of America	740	3,800	81,695.2	69	0	3	4	7	8.93
Uruguay	270	900	22,564.2	73	0	4	4	8	5.42
Uzbekistan	220	500	2,496.1	33	5	4	4	13	2.45
Yemen		300	533.4	16	5	4	4	13	0.02
Zambia	10	300	1,369.1	37	5	4	4	13	3.81
Zimbabwe		400	1,592.4	24	5	4	4	13	3.11

*Note*: Blank spaces indicates that the data were not available.

CPI, Corruption Perceptions Index; GDP, gross domestic product; IV, intravenous; USD, U.S. Dollar.

Data on drug-use prevalence per 100,000 population, including injection drug use and general drug use, were collected using resources from Harm Reduction International, Our World in Data, and Institute of Health Metrics and Evaluation. For territories or countries where data were unavailable, the corresponding entries were left blank.

In addition, information on alcohol consumption per capita was sourced from the Central Intelligence Agency World Factbook^14^ to perform statistical analyses and explore potential correlations between alcohol consumption and drug-use prevalence. Alcohol consumption per capita was included as a covariate to account for its suspected correlation with both injection and illicit drug use, reflecting its potential role as a behavioral indicator of broader substance-use patterns. IRB approval was not required for this study because the analysis was based exclusively on publicly available data and did not involve human participants or animal subjects.

GDPI, developed by the Harm Reduction Consortium, scores countries (0–100) on the basis of their alignment with health, human rights, and development principles in drug policy. It assesses 5 areas: criminal justice, harm reduction, access to medicines, development, and international cooperation. Unlike measures of legal stringency, the GDPI reflects policy quality and implementation. In this study, it was used to compare rights-based policy effectiveness with regulatory strictness captured by the ODPS.

Data on CPI were gathered from Transparency International. The CPI, published annually, assesses public sector corruption in countries and territories worldwide. It scores countries on a scale from 0 to 100, where 0 indicates high corruption, and 100 reflects a clean public sector. The index is derived from expert assessments and surveys, drawing data from reputable sources. It offers insights into global corruption trends, highlights governance challenges, and serves as a valuable tool for policymakers, researchers, and activists.

To address missing values in publicly available international data sets, analyses were conducted using pairwise deletion (a method for handling missing data in which each statistical estimate is calculated using all available cases with complete data for the specific variables involved, allowing sample size to vary across analyses), which allowed to retain the maximum number of observations for each correlation and regression model while acknowledging variability in data completeness across countries.

All the collected data are compiled in Table 1 for reference, analysis, and public use. To quantify the stringency of drug policies, open sources were utilized, including the European Monitoring Centre for Drugs and Drug Addiction, the European Union Drug Agency, the United Nations Office on Drugs and Crime, International Narcotics Control Board, National Drug and Alcohol Research Centre, Global Commission on Drug Policy, and official governmental publications of legislature from the respective countries (Appendix Table 1, available online). The data collected from these sources were analyzed and evaluated the respective countries on the basis of their legislative frameworks, utilizing the detailed scale provided below. The evaluation focused on the stringency of laws governing cannabis, stimulants and hallucinogens, and injection drugs. The information was collated, and specific scores were assigned to each drug group in accordance with the scale, allowing for a quantification of legislative strictness across different substances (Table 1).

### Measures

The stringency of cannabis regulation was assessed using the following scale: 0, cannabis is legal for both medicinal and recreational use; 1, medicinal use is legal, and recreational use is not criminalized; 2, recreational use is decriminalized; 3, recreational or medicinal use is criminalized, but only fines are imposed, same rules apply for possession of small amount; 4, possession of small amount of cannabis may result in jail time; and 5, consumption of cannabis may lead to jail time, or there is a death penalty for possession, use, distribution, or production.

The stringency of hallucinogen (ecstasy, ketamine, psilocybin, and others) and stimulant (amphetamines, cocaine, and others) regulation was assessed using the following scale: 0, legal; 1, personal use is decriminalized; 2, personal use is criminalized, however, only fines are imposed for possession of small amount or personal use; 3, possession of small amount may result in jail time; and 4, consumption of these drugs may lead to jail time, or there is a death penalty for possession, use, distribution, or production, or there is a death penalty for possession, use, distribution, or production.

The stringency of IV drug (heroin, morphine, others) regulation was assessed using the following scale: 0, legal; 1, personal use is decriminalized; 2, personal use is criminalized, however, only fines are imposed for possession of small amount or personal use; 3, possession of small amount may result in jail time; and 4, consumption of these drugs may lead to jail time, or there is a death penalty for possession, use, distribution, or production, or there is a death penalty for possession, use, distribution, or production.

ODPS (Chronbach’s α=0.83) for each country was calculated by summing the individual scores from cannabis, hallucinogen/stimulant, and IV drug regulations, reflecting the comprehensive stringency of national drug policies. Whereas the ODPS developed in this study quantifies the stringency of drug legislation—focusing on the legal status and penalties associated with cannabis, hallucinogens, stimulants, and IV drugs—GDPI offers a broader, multidimensional assessment of national drug policies. Unlike ODPS, which centers on the severity of legal sanctions, GDPI evaluates how well a country aligns its drug policy with international norms related to health, human rights, harm reduction, and development goals. Therefore, ODPS captures the restrictiveness of a country’s legal framework, whereas GDPI measures the quality and humanity of drug governance.

### Statistical Analysis

Statistical analyses were performed using SPSS, Version 26, a software program commonly used for data management and statistical testing. Pearson’s correlation was applied, which measures the strength and direction of the relationship between 2 continuous variables, as well as bivariate and multivariate linear regression analyses, which evaluate how 1 or more independent variables predict changes in an outcome variable. These methods were used to examine associations among GDP per capita, corruption index, alcohol consumption, and drug-use prevalence. Mean values, 95% CIs, and *p*-values were reported, with *p*<0.05 considered statistically significant.

## RESULTS

**Figure 1:**
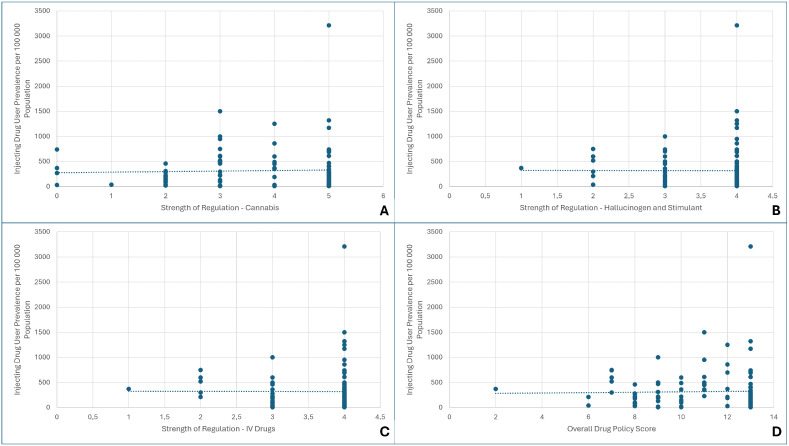
A linear relationship between the prevalence of people who use injection drugs and the strength of regulation score for cannabis, hallucinogen and stimulant drugs, intravenous drugs, and ODPS. (A) Cannabis. (B) Hallucinogen and stimulant drugs. (C) Intravenous drugs. (D) ODPS. *Note*: This figure illustrates linear associations between injection drug-use prevalence and drug regulation strength. No association was detected. ODPS, overall drug policy score.

The data indicate that there are no statistically significant correlations between IDU prevalence and the regulation strength of cannabis, hallucinogens and stimulants, IV drugs, or the ODPS (Table 2 and Figure 1). There was also no correlation between GDPI and IDU (Table 2). The data indicate that there is a statistically significant weak positive correlation between pure alcohol consumption per capita and IDU prevalence (Table 2 and Appendix Figure 1, available online).

**Table 2:** Data From Linear Regression and Pearson's Correlation Analyses

Regulations	Injecting drug-user prevalence	Illicit drug-user prevalence
Regulation strength (cannabis)	R (108)=0.03, *p*=0.35	R (179)= −0.40, *p*<0.001
	R squared=0.001, *p*=0.70	R squared=0.16, *p*<0.001
	F=0.15, *p*=0.70	F=34.88, *p*<0.001
	Beta=10.92 (95% CI= −45.38, 67.22), *p=*0.70	Beta= −124.99 (95% CI= −166.75, −83.23), *p*<0.001
Regulation strength (hallucinogens and stimulants)	R (108)= −0.003, *p*=0.48	R (179)= −0.29, *p*<0.001
	R squared=0.00, *p*=0.98	R squared=0.09, *p*<0.001
	F=0.001, *p*=0.98	F=16.89, *p*<0.001
	Beta= −1.99 (95%= −124.60, 120.64), *p*=0.98	Beta= −206.09 (95% CI= −305.04, −107.14), *p*<0.001
Regulation strength (intravenous drugs)	R (108)= −0.004, *p*=0.48	R (179)= −0.19, *p*<0.01
	R squared=0.00, *p*=0.97	R squared=0.04, *p*=0.01
	F=0.002, *p*=0.97	F=6.57, *p*=0.01
	Beta= −2.67 (95% CI= −132.26, 126.92), *p*=0.97	Beta= −138.73 (95% CI= −245.51, −31.94)
Overall drug policy score	R (108)=0.02, *p*=0.42	R (179)= −0.38, *p*<0.001
	R squared=0.00, *p*=0.83	R squared=0.14, *p*<0.001
	F=0.046, *p*=0.83	F=29.73, *p*<0.001
	Beta=3.70 (95% CI= −30.57, 37.97), *p*=0.83	Beta= −71.98 (95% CI= −93.03, −45.93), *p*<0.001
GDP per capita	R (108)=0.15, *p*=0.06	R (179)=0.60, *p*<0.001
	R squared=0.02, *p*=0.13	R squared=0.36, *p*<0.001
	F=2.32, *p*=0.13	F=99.91, *p*<0.001
	Beta=0.002 (95% CI= −0.001, 0.005), *p*=0.13	Beta=0.01 (96% CI=0.008, 0.012), *p*<0.001
Corruption perceptions index	R (106)=0.25, *p*<0.01	R (162)=0.63, *p*<0.001
	R squared=0.06, *p*<0.01	R squared=0.40, *p*<0.001
	F=7.23, *p*<0.01	F=107.50, *p*<0.001
	Beta=0.002 (95% CI= −0.001, 0.005), *p*<0.01	Beta=14.24 (95% CI=11.53, 16.96), *p*<0.001
Pure alcohol per capita	R (103)=0.32, *p*<0.001	R (167)=0.54, *p*<0.001
	R squared=0.10, *p*=0.001	R squared=0.29, *p*<0.001
	F=11.71, *p*=0.001	F=61.50, *p*<0.001
	Beta=33.62 (95% CI=14.13, 53.11), *p*=0.001	Beta=33.62 (96% CI=46.91, 76.09), *p*<0.001
Democracy index	R (103)=0.14, *p*=0.076	R (150)=0.55, *p*<0.001
	R squared=0.02, *p*=0.153	R squared=0.30, *p*<0.001
	F=2.07, *p*=0.153	F=66.53, *p*<0.001
	Beta – 16.75 (95% CI= −6.33, 39.84), *p*<0.001	Beta=102.96 (95% CI=78.02, 127.91), *p*<0.001
Global Drug Policy Index	R (22)=0.22, *p*=0.151	R (22)=0.48, *p*=0.005
	R squared=0.05, *p*=0.151	R squared=0.20, *p*=0.005
	F=1.21, *p*=0.301	F=7.57, *p*=0.011
	Beta=4.79 (95% CI= −4.60, 14.20), *p*=0.301	Beta=19.36 (96% CI=4.90, 33.83), *p*=0.11
Illicit drug-user prevalence	R (103)=0.28, *p*=0.001	
	R squared=0.08, *p*=0.003	
	F=9.26, *p*=0.003	
	Beta=0.24 (95% CI=0.08, 0.39), *p*=0.003

The data also indicate that there is no statistically significant correlation between IV drug-use prevalence and GDP per capita and DI (Table 2 and Appendix Figure 2, available online). However, there is statistically significant weak positive correlation between IV drug user prevalence and CPI (Table 2 and Appendix Figure 3, available online).

**Figure 2:**
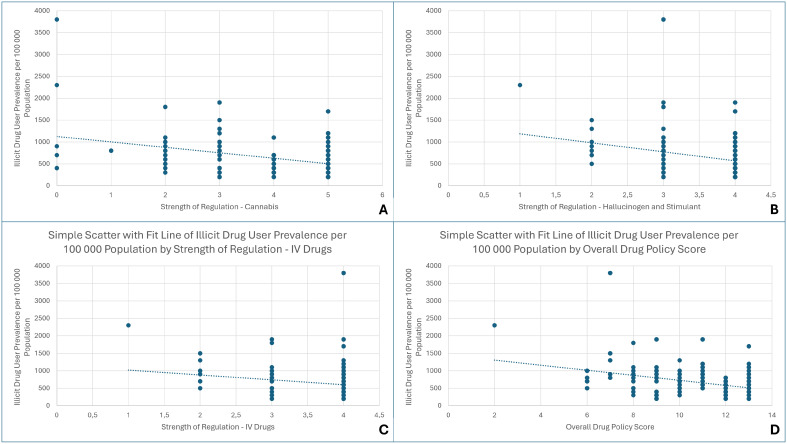
A linear relationship between the prevalence of people who use illicit drugs and the strength of regulation score for cannabis, hallucinogen and stimulant drugs, intravenous drugs, and ODPS. (A) Cannabis. (B) Hallucinogen and stimulant drugs. (C) Intravenous drugs. (D) ODPS. *Note*: This figure depicts the linear relationships between the prevalence of illicit drug use and regulation strength scores across 4 drug categories. Panel A shows a weak negative correlation with cannabis regulation strength, Panel B shows a similar weak negative trend for hallucinogen and stimulant drug regulation, Panel C reflects a weak inverse relationship with intravenous drug regulation, and Panel D reveals weak negative association between ODPS regulation strength and illicit drug use prevalence. ODPS, overall drug policy score.

The data indicate that there are statistically significant weak negative correlations between ILDU prevalence and the regulation strength of cannabis, hallucinogens and stimulants, IV drugs, or the ODPS (Table 2 and Figure 2). There was a statistically significant moderate positive correlation between GPDI and ILDU (Table 2).

The data indicate that there is a statistically significant moderate positive correlation between pure alcohol consumption per capita and ILDU prevalence (Table 2 and Appendix Figure 4, available online). The data indicated that there is statistically significant moderate positive correlation between GDP per capita, CPI, DI, and ILDU prevalence (Table 2 and Appendix Figures 5 and 6, available online). The data indicate that there is statistically significant weak positive correlation between ILDU and IDU prevalence (Table 2 and Appendix Figure 7, available online).

The data indicate that there are statistically significant weak negative correlations between CPI and the regulation strength of cannabis (r [163]= −0.26, *p*<0.01), hallucinogens and stimulants (r [163]= −0.22, *p*<0.01), regulation strength of IV drugs (r [163]= −0.15, *p*=0.03), and the ODPS (r [163]= −0.26, *p*<0.001) (Appendix Figure 8, available online). Given the weak to moderate positive correlation between drug-use prevalence and CPI, the authors categorized countries into 2 groups—60+ (less corrupt) and <60 (more corrupt)—to compare the means (Table 3 and Appendix Figures 9 and 10, available online). The differences in means were statistically significant for both IDU prevalence (95% CI=76.26, 431.98; *p*<0.01) and illicit drug-use prevalence (95% CI=520.65, 782.19; *p*<0.001) (Table 3 and Appendix Figure 10, available online).

**Table 3:** Means for Less and More Corrupt Countries in Regard to Injecting Drug-User Prevalence and Illicit Drug-User Prevalence

Corruption level	Mean	*n*	SD
Injecting drug-user prevalence per 100,000 population			
Less corrupt countries	502.50	24	629.584
More corrupt countries	248.38	84	286.389
All countries	304.85	108	400.113
Illicit drug-user prevalence per 100,000 population			
Less corrupt countries	1,146.88	32	626.812
More corrupt countries	495.45	132	216.094
All countries	622.56	164	423.443

For ILDU prevalence, in the multivariate analysis including DI, CPI, ODPS, GDP per capita, alcohol consumption per capita, and the GDPI, the overall model accounted for 66% of the variance in ILDU prevalence (R[19]=0.81, R²=0.66, *p*=0.001). However, none of the individual predictors demonstrated statistically significant associations within the model, except for alcohol consumption (*p*=0.032). In the stepwise regression procedure, none of the candidate predictors met the entry criterion, and therefore, all variables were excluded from the model. Variance inflation factor (VIF) analysis showed VIF values of 3.98 for GDP per capita, 10.21 for the CPI, 4.10 for the DI, 1.35 for alcohol consumption per capita, and 2.72 for the GDPI, indicating acceptable multicollinearity for most predictors, whereas the CPI displayed notably elevated VIF, suggesting considerable overlap with other governance-related variables in the model.

For IDU prevalence, in the corresponding multivariate model for IDU prevalence, which included the same 6 predictors, the model explained 61% of the variance (R[15]=0.78, R²=0.61; *p*=0.015). Within this model, only DI (*p*=0.016) and alcohol consumption per capita (*p*=0.006) remained statistically significant. Stepwise regression retained only alcohol consumption per capita as a predictor of injection drug use prevalence (β=0.32, *p*=0.001). All other variables (ODPS, GDP per capita, CPI, and DI) were excluded by the model. The final model explained 10% of the variance (R²=0.01, *p*=0.001). VIF analysis showed VIF values of 4.41 for GDP per capita, 14.14 for the CPI, 4.80 for the DI, 1.37 for alcohol consumption per capita, and 3.67 for the GDPI, indicating that multicollinearity was generally acceptable for most predictors but substantially elevated for the CPI, suggesting overlap with other governance-related variables in the model.

The analysis of variance did not detect any statistically significant relationship between the strictness of drug legislation and an increase in IDU prevalence in regard to both ODPS and GDPI. However, a statistically significant relationship was found between pure alcohol consumption and prevalence, explaining approximately 10% of variance (ANOVA). A smaller but still significant relationship was identified between ILDU prevalence and IDU prevalence (ANOVA: 8%). GDP per capita did not correlate significantly with IDU prevalence, whereas the CPI did, accounting for 6% of variance. Multivariate analysis found that only DI and alcohol consumption retained their statistical significance. Although in bivariate analysis, there was no statistically significant association between DI and IDU.

Regarding ILDU prevalence, the strongest correlation was observed with CPI, indicating that less corrupt countries had higher prevalence rates (ANOVA: 40%). GDP per capita was the second strongest factor, explaining 36% of variance—higher GDP was associated with higher prevalence. Pure alcohol consumption accounted for 29% of variance, with higher alcohol intake correlating with increased ILDU prevalence. Correlations between ILDU prevalence and regulation strength were considerably weaker, ranging from 4% to 16% in explained variance, with the strongest negative correlation identified with the strictness of cannabis regulation; however, GDPI showed moderate positive correlation with ANOVA of 20%. Multivariate analysis did not find any of the factors significant. Multivariate analysis did not find either GDPI or ODPS to be a statistically significant predictor for IDU and ILDU.

## DISCUSSION

This analysis found no evidence that the strictness of drug legislation, as measured by either the ODPS or the GDPI, was associated with higher or lower prevalence of IDU. Instead, behavioral and contextual factors emerged as more consistent correlates. Alcohol consumption demonstrated a statistically significant association with IDU prevalence, accounting for approximately 10% of the variance, whereas a modest but significant overlap was also observed between ILDU and IDU prevalence. Although GDP per capita did not correlate with IDU, the CPI showed a weak but significant relationship, explaining around 6% of the variance. In the multivariate model, only DI and alcohol consumption retained statistical significance, despite the absence of a bivariate association between DI and IDU.

For ILDU prevalence, the strongest relationships were observed with governance and economic indicators rather than regulatory stringency. Less corrupt countries reported higher prevalence, with CPI explaining 40% of the variance, whereas GDP per capita accounted for 36%, and alcohol consumption explained 29%. In contrast, the correlations between ILDU and regulation strength were weaker (4%–16%), with the only notable pattern being a negative association with cannabis regulation. Interestingly, GDPI demonstrated a moderate positive correlation with ILDU prevalence, suggesting that rights-based drug policies may be associated with greater reported use. However, when entered into multivariate models, only alcohol consumption remained statistically significant.

An important consideration in interpreting the association between the CPI and the GDPI is the role of reporting quality and security contexts. Countries with lower CPI scores (i.e., more corrupt systems) often face greater security challenges, weaker institutional transparency, and less patient-centered health systems. In such settings, individuals may be more reluctant to disclose drug use owing to fear of legal consequences, stigma, or distrust of authorities, which can lead to systematic underreporting. Conversely, less corrupt countries with stronger patient-driven approaches and more transparent governance structures are more likely to generate accurate prevalence estimates. This dynamic may partly explain why higher CPI and GDPI values are associated with greater reported drug use, not necessarily because rights-based or less corrupt environments increase actual prevalence but because they foster conditions where data collection is more reliable, and individuals are more willing to report sensitive behaviors. This potential underreporting bias should therefore be recognized as a limitation of the study.

Cannabis, tobacco, and alcohol are often labeled as gateway drugs.^6^ Multivariate analysis found alcohol to be a significant predictor for IDU. Taking both data analysis and the existing literature into account, there is no rational explanation for why alcohol is legal in many parts of the world, whereas cannabis remains prohibited in the most regions.

Ultimately, the question of why these findings matter lies in what they reveal about the limits of traditional drug regulation. The analysis shows that neither stringent legislation nor rights-based policy quality meaningfully predicts national patterns of drug use, whereas behavioral and governance factors—particularly alcohol consumption, economic context, and corruption levels—demonstrate much stronger associations. This suggests that global debates focused primarily on criminalization, decriminalization, or legislative reform may be overlooking the deeper drivers of drug use. Therefore, what matters is that strengthening or relaxing drug laws alone is unlikely to reduce prevalence; policies must instead address the social, cultural, and behavioral environments in which substance use occurs. These results reinforce the need to shift from enforcement-centered frameworks toward comprehensive public-health strategies that target underlying risk structures rather than relying on the legal system to shape population-level behavior.

### Limitations

This study has several limitations that should be considered when interpreting the findings. First, the cross-sectional design restricts the ability to infer causality between drug policy stringency and drug-use prevalence. Second, reliance on publicly available data sets introduces variability in data quality, completeness, and reporting standards across countries, with some nations lacking data altogether. Third, the accuracy of prevalence estimates may be compromised in highly corrupt or repressive contexts, where underreporting is likely due to stigma or fear of legal consequences. Although ODPS offers a novel approach to quantifying legal stringency, it simplifies complex legal systems and does not account for enforcement practices or regional variation within countries.

In conclusion, despite significant financial investments in drug enforcement, the war on drugs has resulted in serious consequences, including black market activity, violence, and corruption. Multivariate data analysis indicates that the strength of regulation is not a statistically significant predictor of drug-use prevalence. Furthermore, global data suggest that drug-user prevalence is on a rise.^1^, ^2^, ^3^ Combined with evidence from Portugal and Switzerland, this suggests that health-centered approaches may be more effective, raising the question of whether drug policy should prioritize reducing overall drug-use prevalence or instead focus on minimizing drug-related harms and harmful patterns of use.

## CONCLUSIONS

In conclusion, whereas bivariate analyses suggested several associations between national characteristics and drug-use prevalence, multivariate analysis revealed that neither the GDPI nor the ODPS significantly predicted injection or illicit drug-use prevalence. Only alcohol consumption per capita and DI emerged as consistent predictors for injection drug use, and only alcohol emerged as that of illicit drug use. These findings suggest that the stringency or rights-based orientation of drug laws alone may not sufficiently explain patterns of substance use across countries. Instead, behavioral, cultural, and social determinants such as alcohol use and general substance involvement may play a more direct role. This underscores the complexity of global drug-use dynamics and highlights the need for multifaceted, evidence-based health-centered strategies that go far beyond just prohibitive legislative frameworks. Future research should consider incorporating more granular data, enforcement practices, and longitudinal designs to better understand how policy interacts with real-world behavior.

## CRediT AUTHOR STATEMENT

Konstantine Chakhunashvili: Conceptualization, Methodology, Formal analysis, Supervision, Writing – original draft, Writing – review & editing. Giorgi Meladze: Conceptualization, Methodology, Writing – review & editing. Eka Kvirkvelia: Investigation, Writing – review & editing. Davit G. Chakhunashvili: Methodology, Formal analysis, Writing – review & editing.

## Appendix Supplementary materials

Image, image 1 dummy alt text

Image, image 2 dummy alt text

Image, image 3 dummy alt text

Image, application 4

Image, image 5 dummy alt text

Image, image 6 dummy alt text

Image, image 7 dummy alt text

Image, image 8 dummy alt text

Image, image 9 dummy alt text

Image, image 10 dummy alt text

Image, image 11 dummy alt text

## Footnotes

Supplementary material associated with this article can be found in the online version at doi:10.1016/j.focus.2026.100499.
