# Supplementary figures and images for: Evaluating the Impact of Drug Regulation Stringency on Global Drug-Use Patterns: A Cross-Sectional Study
Source: AJPM Focus. 2026 Apr 1;5(4):100499. doi: 10.1016/j.focus.2026.100499 (PMC13311835; doi:10.1016/j.focus.2026.100499)

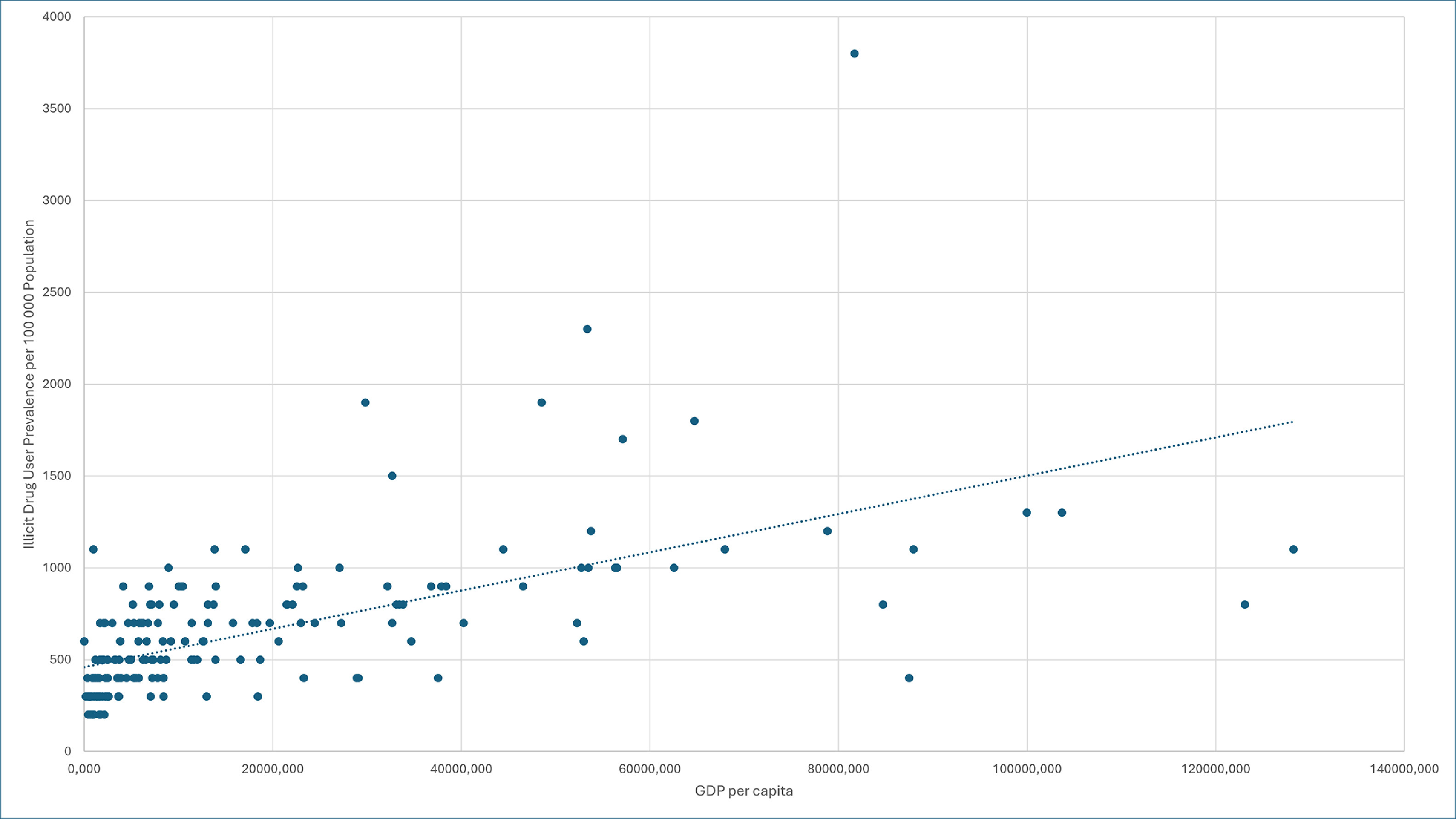

Supplement: Supplementary file 1 [file mmc1.jpg]

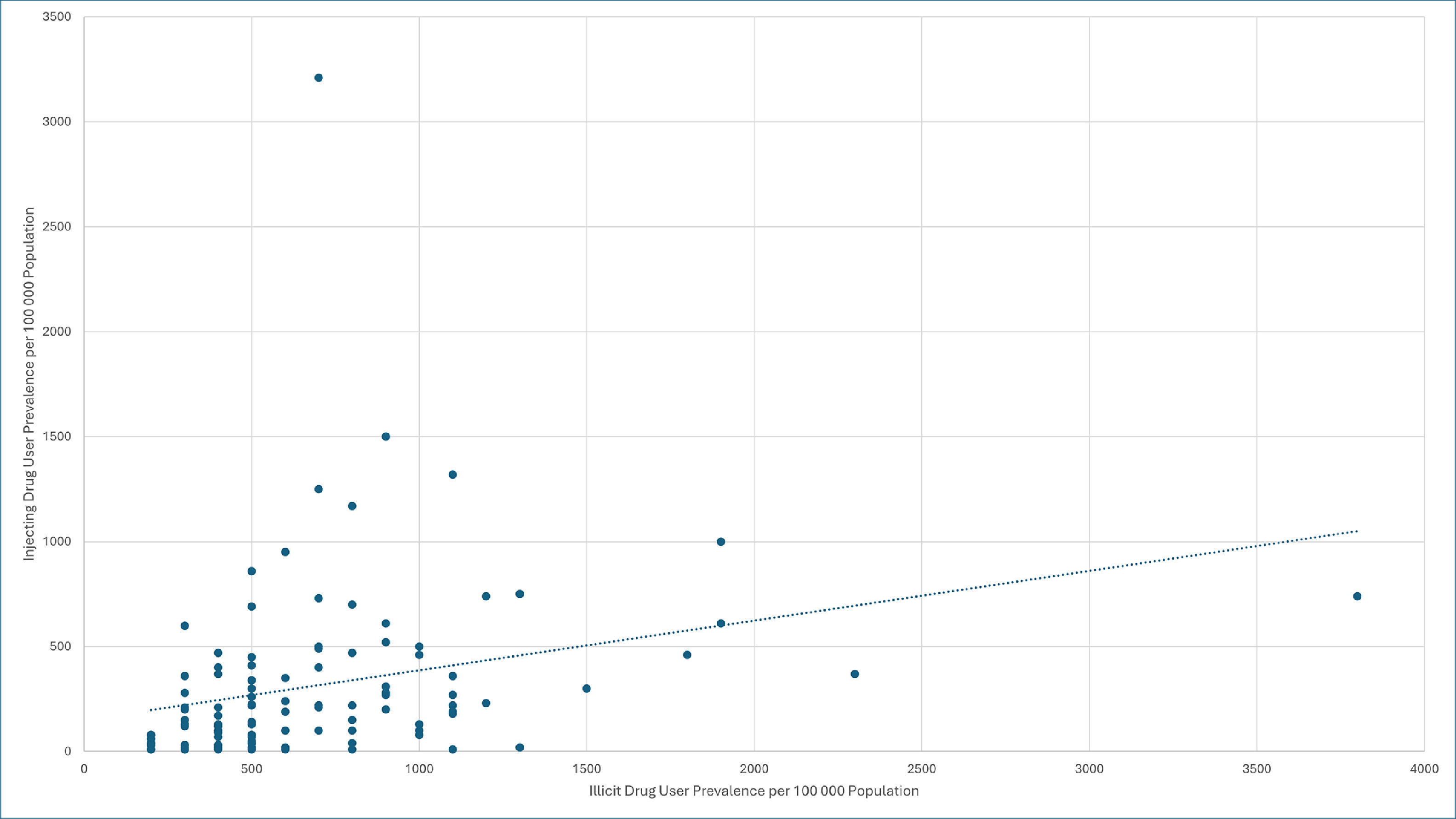

Supplement: Supplementary file 2 [file mmc2.jpg]

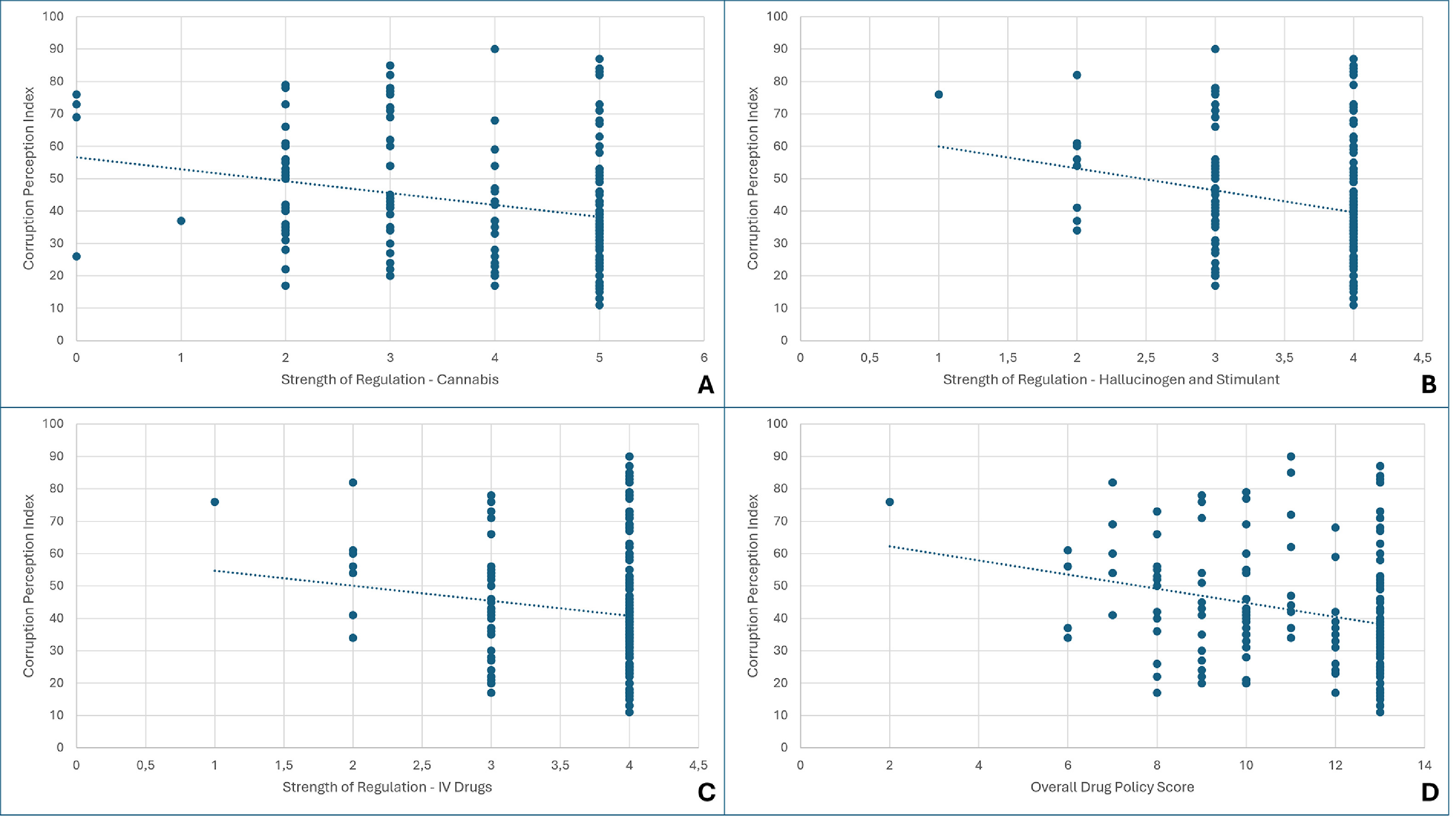

Supplement: Supplementary file 3 [file mmc3.jpg]

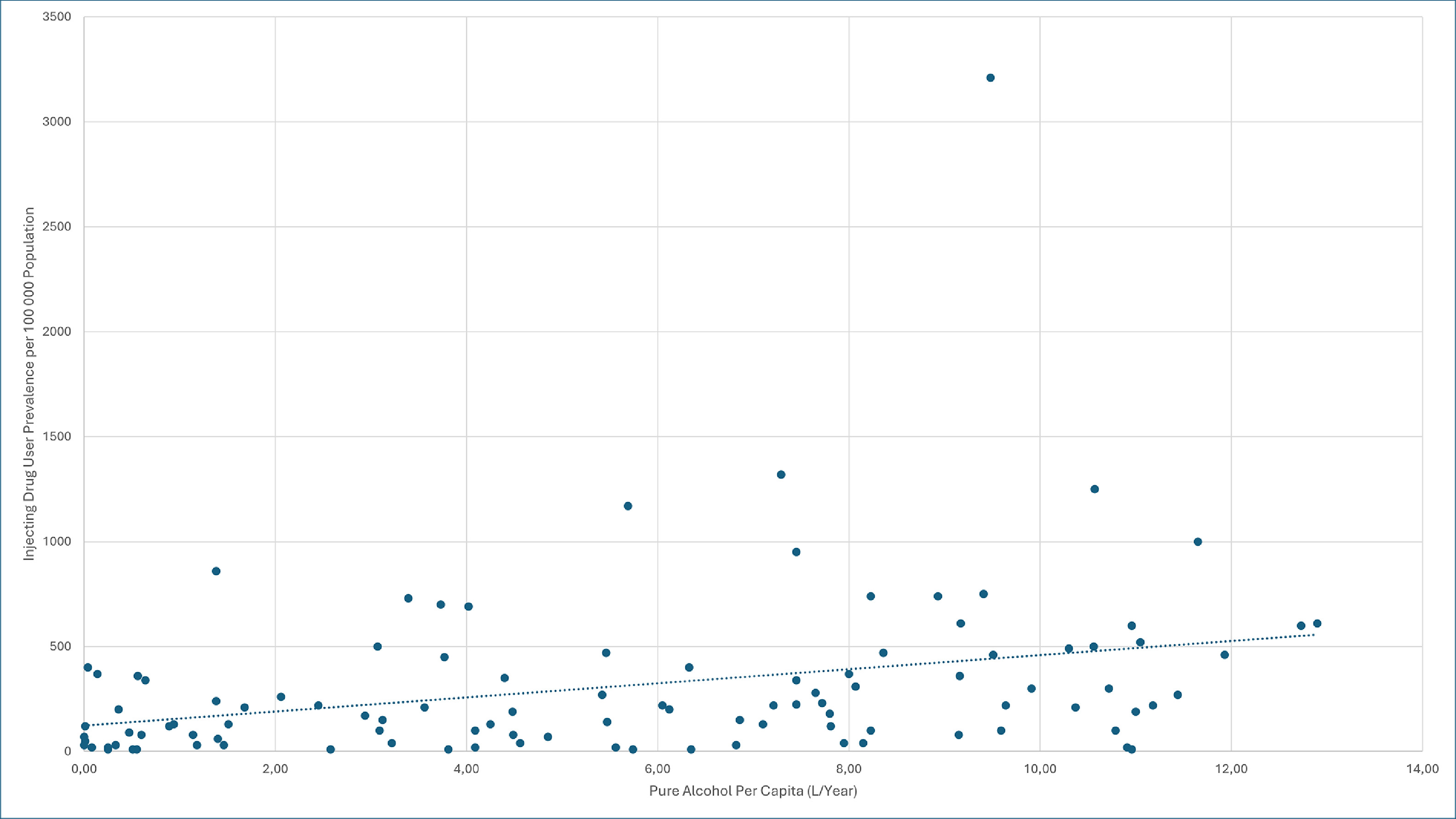

Supplement: Supplementary file 5 [file mmc5.jpg]

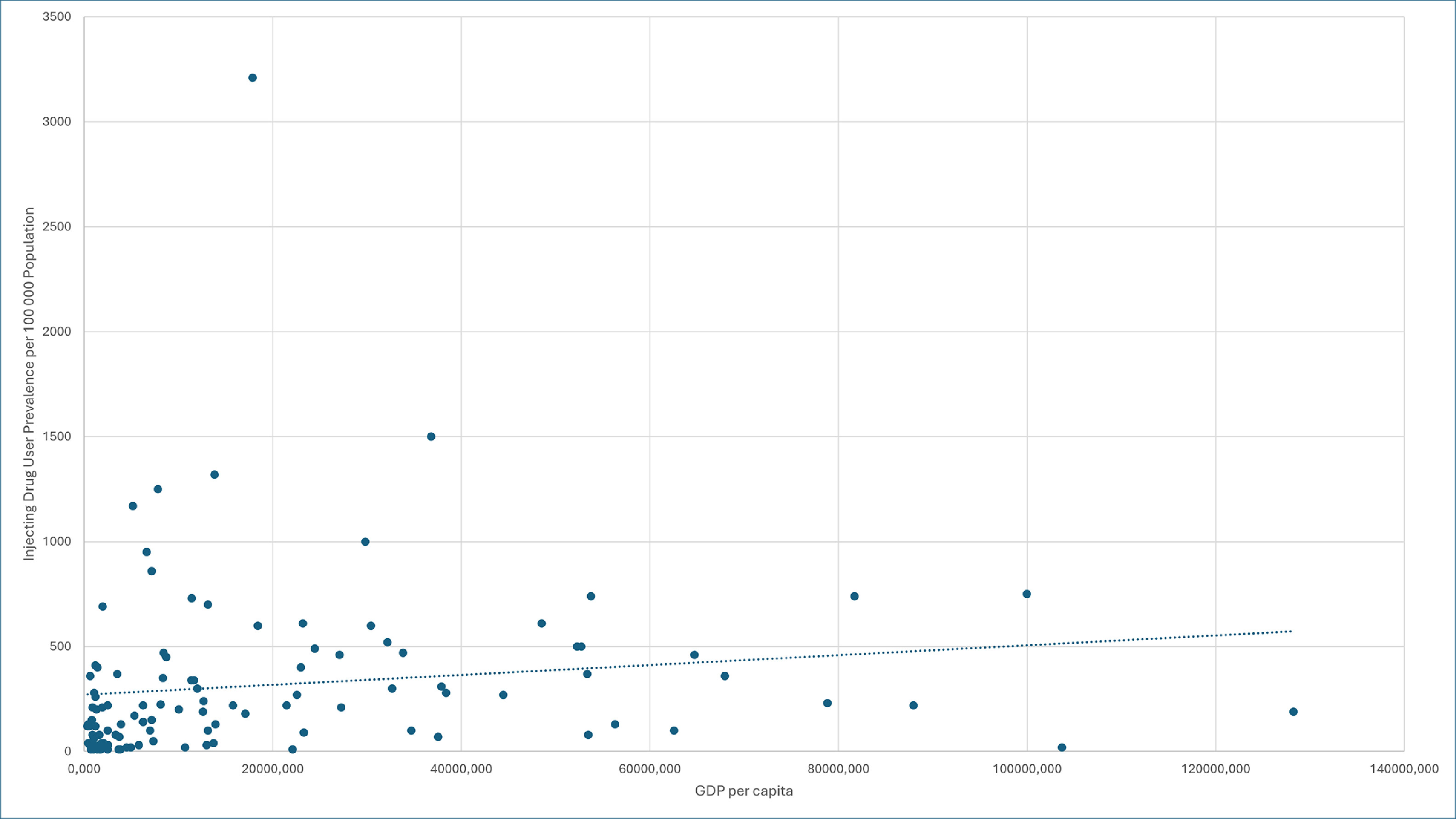

Supplement: Supplementary file 6 [file mmc6.jpg]

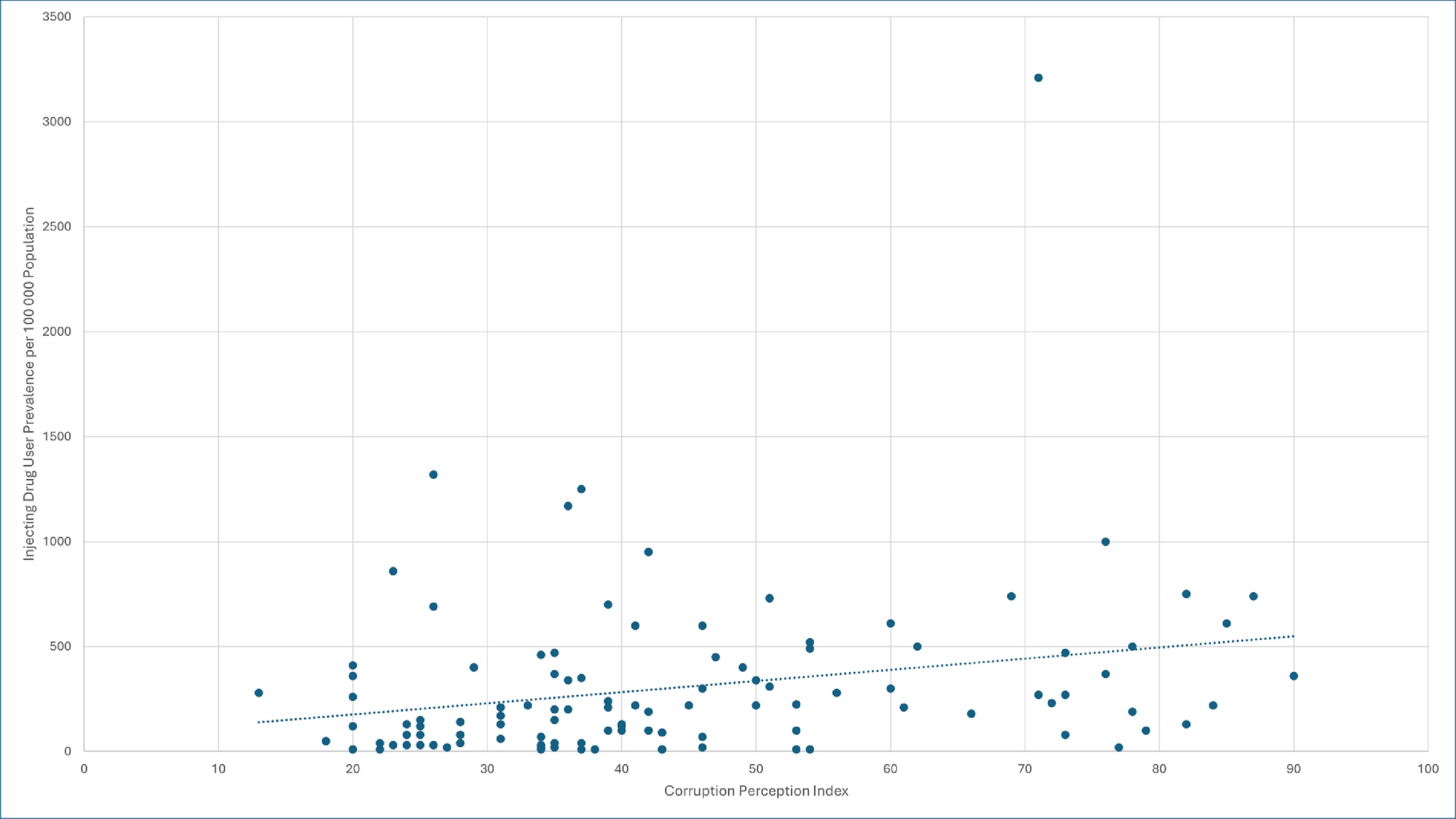

Supplement: Supplementary file 7 [file mmc7.jpg]

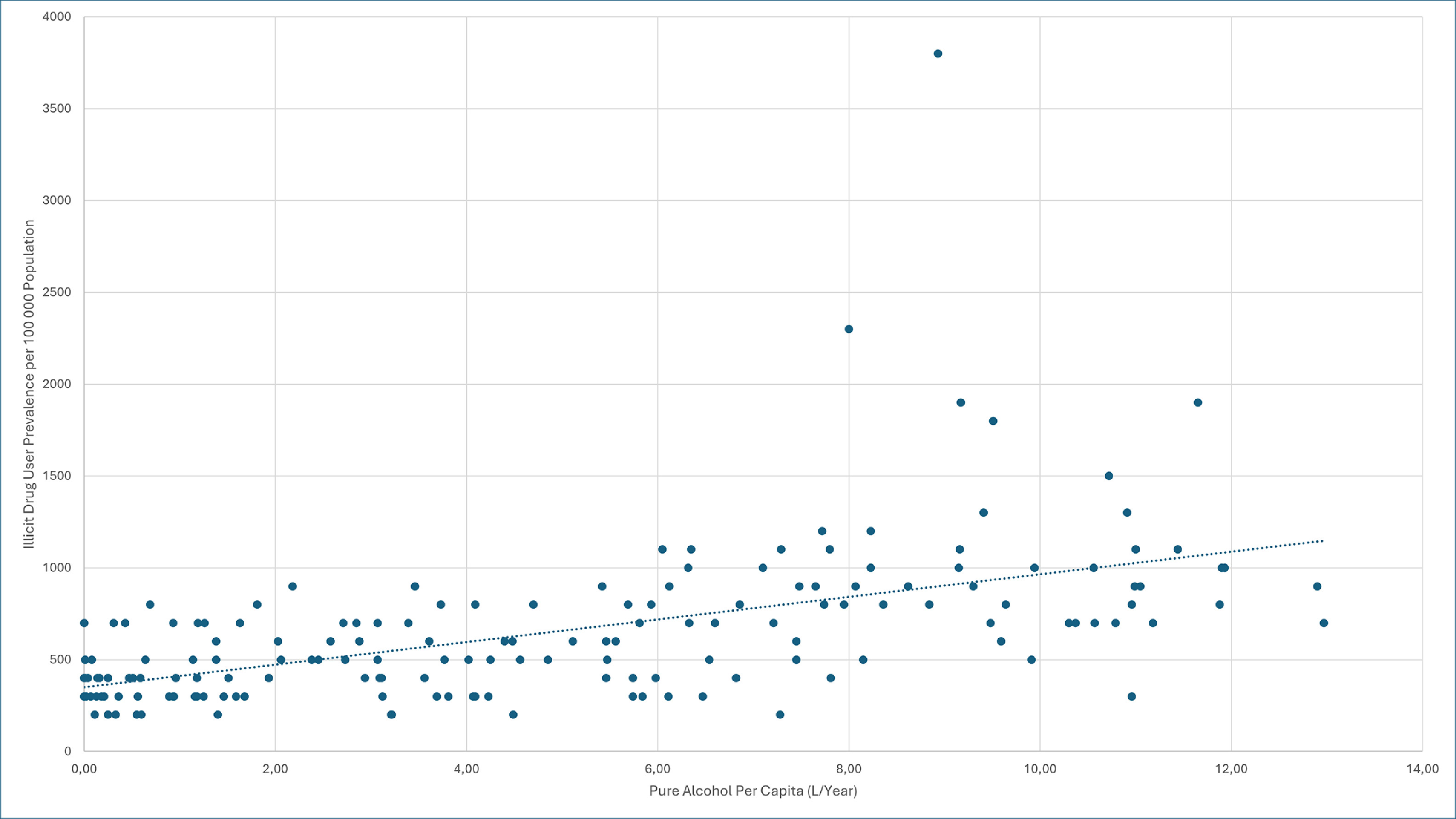

Supplement: Supplementary file 8 [file mmc8.jpg]

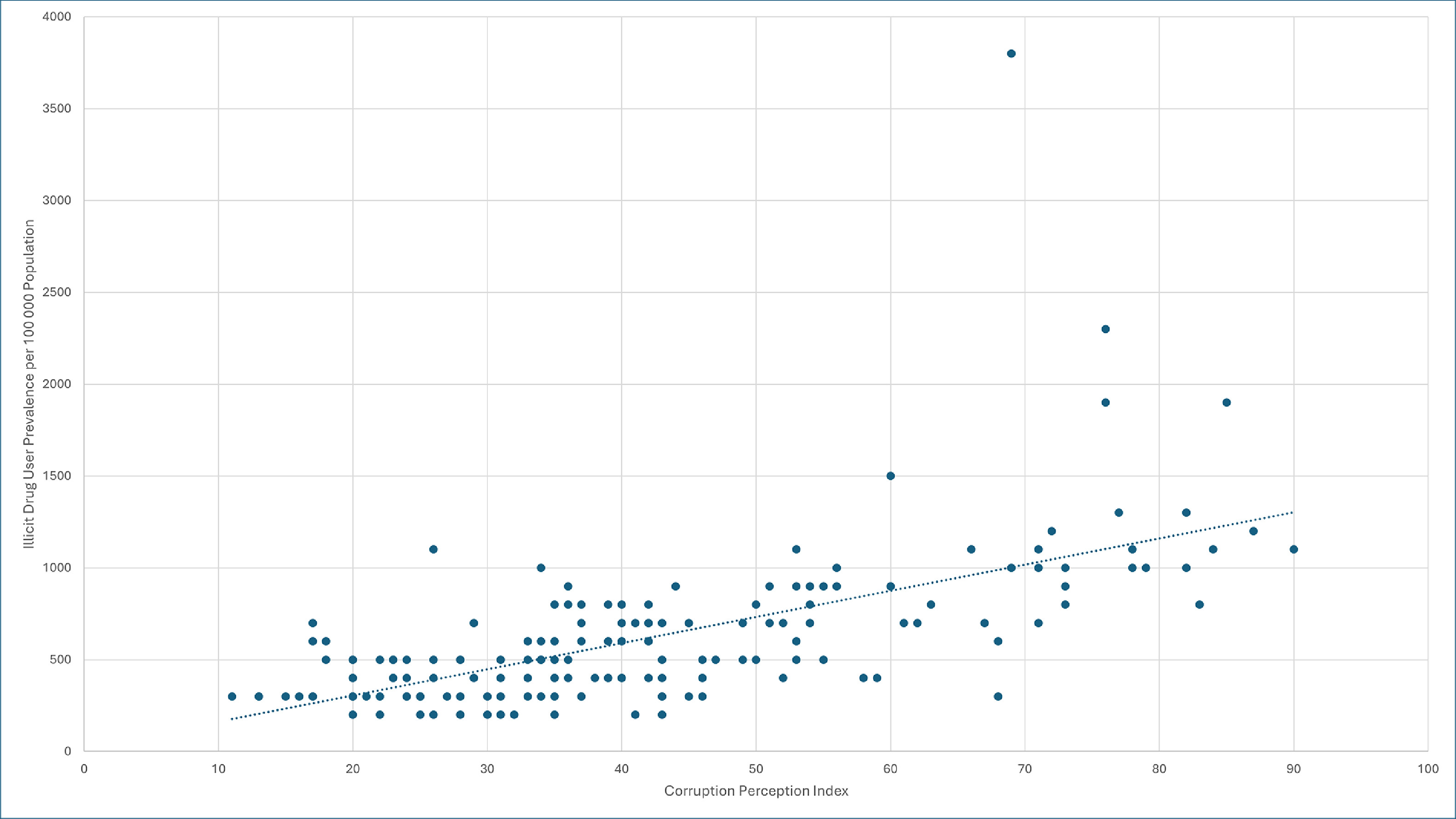

Supplement: Supplementary file 9 [file mmc9.jpg]

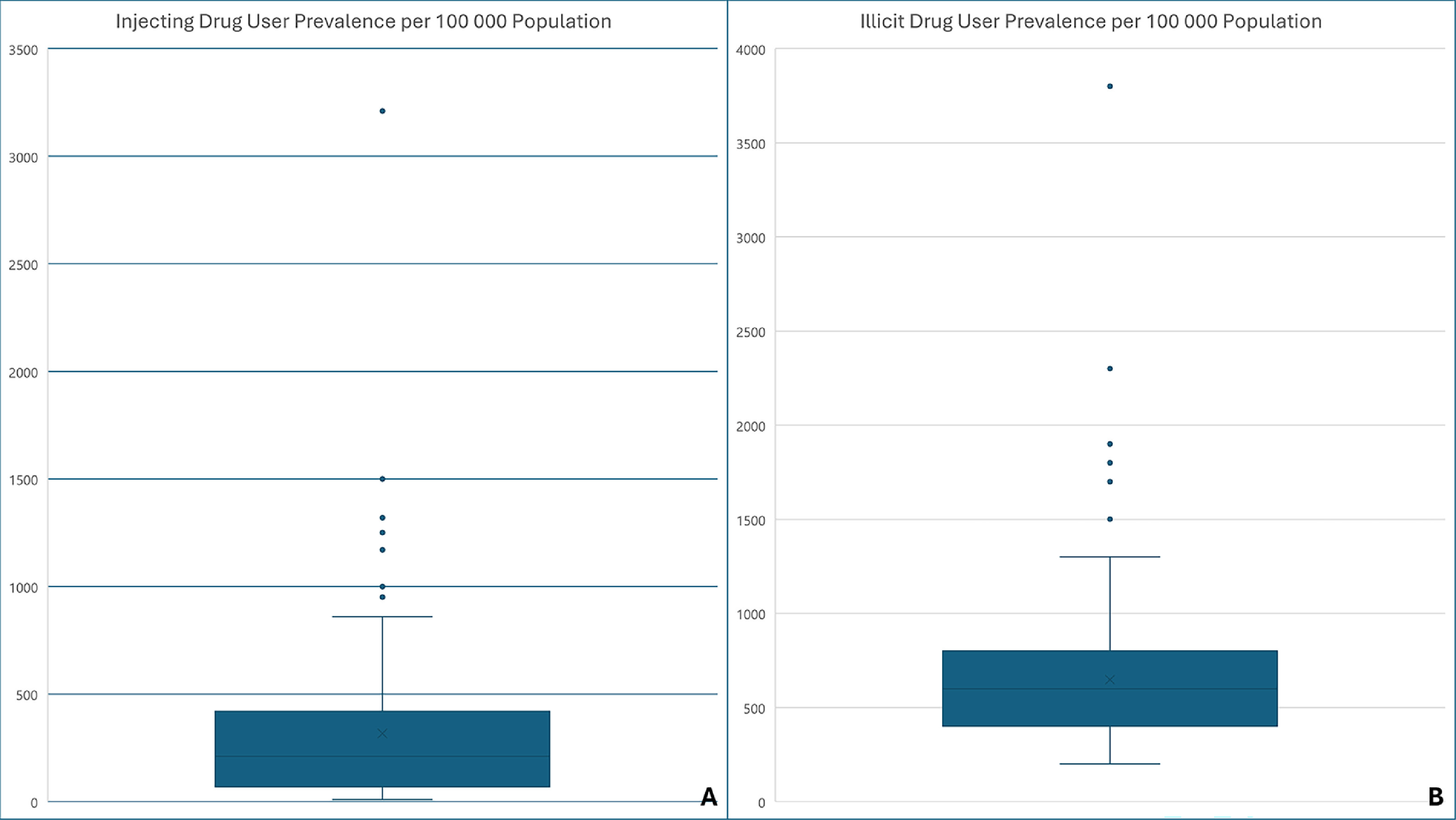

Supplement: Supplementary file 10 [file mmc10.jpg]

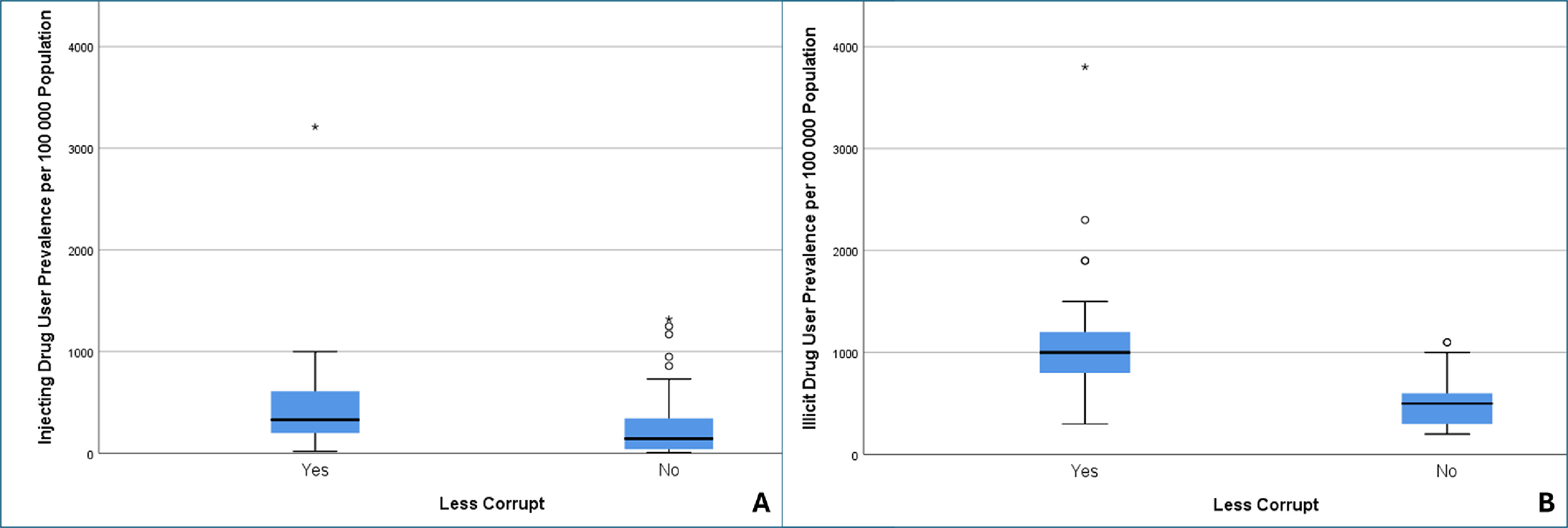

Supplement: Supplementary file 11 [file mmc11.jpg]
