# Supplementary material for: Evaluating the Impact of Drug Regulation Stringency on Global Drug-Use Patterns: A Cross-Sectional Study
Source: AJPM Focus. 2026 Apr 1;5(4):100499. doi: 10.1016/j.focus.2026.100499 (PMC13311835; doi:10.1016/j.focus.2026.100499)
Supplement: Supplementary file 4 [file mmc4.docx]

Supplemental Material 1. This supplemental file contains all the sources from which the stringency data was collected.

References:

1. NCDAS. Drug Abuse Statistics. 2024.
2. Rauschert C, Möckl J, Seitz NN, Wilms N, Olderbak S, Kraus L. The Use of Psychoactive Substances in Germany: Findings from the Epidemiological Survey of Substance Abuse 2021. Dtsch Arztebl Int. 2022;119:527-34.
3. Afghanistan. Law on Campaign against Intoxicants, Drugs and their Control. Ministry of Justice, Islamic Republic of Afghanistan; 2015 Nov 15. Available from: <https://laws.moj.gov.af/en/>
4. Albania. Law on Narcotic Drugs and Psychotropic Substances, Law No. 7975. Official Publishing Center, Republic of Albania; 1995 Jul 26. Available from: <https://qbz.gov.al/>
5. Algeria. Law No. 04-18 of 13 Dhou El Kaada 1425 (25 December 2004), Relative to the Prevention and Repression of the Illicit Use and Trafficking of Narcotics and Psychotropic Substances. Official Gazette of the People's Democratic Republic of Algeria; 2004 Dec 25. Available from: <https://www.unodc.org/res/cld/document/dza/loi-04-18_html/algeria-loi04-18.pdf>
6. American Samoa. American Samoa Code Annotated - Title 13, Chapter 10. Section 13.1022 - Possession of Controlled Substance Unlawful. Available from: <https://asbar.org/code-annotated/13-1022-possession-of-controlled-substance-unlawful/>
7. Andorra. Butlletí Oficial del Principat d'Andorra (BOPA). Official Bulletin of the Principality of Andorra. Available from: <https://www.bopa.ad/Documents?search=drogues>
8. Antigua and Barbuda. Misuse of Drugs Act (Cap. 283). Laws of Antigua and Barbuda; 1974 Jan 24 [amended 2019]. Available from: <https://laws.gov.ag/wp-content/uploads/2018/08/cap-283.pdf>
9. Argentina. Misuse of Drugs Act (Law No. 23.737). 1989; [amended 2019]. Available from: <https://www.unodc.org/LSS/Country/DetailsLegalSystem?code=DLIL&country=AR>
10. Armenia. Law on Narcotic Drugs and Psychotropic (Psychoactive) Substances. National Assembly of the Republic of Armenia; 2002 Dec 26 [amended 2022 Jun 27]. Available from: <https://www.parliament.am/law_docs/100203HO518eng.pdf>
11. Australia. Narcotic Drugs Act 1967. Federal Register of Legislation; 1967. Available from: <https://www.legislation.gov.au/Details/C2021C00198>
12. Australia. Criminal Code Act 1995. Federal Register of Legislation; 1995. Available from: <https://www.legislation.gov.au/Details/C2023C00177>
13. Austria. Suchtmittelgesetz (Narcotic Substances Act). Bundesgesetzblatt für die Republik Österreich; 1998. Available from: <https://www.ris.bka.gv.at/>
14. Azerbaijan. Law on the Control of Illicit Trafficking in Narcotic Drugs, Psychotropic Substances, and Precursors. 2005 Jun 28 [amended 2024 Jun 28]. Available from: <https://sherloc.unodc.org/cld/uploads/res/document/law-on-the-control-of-illicit-trafficking-of-naroctic-drugs-psychotropic-substances-and-precursors_html/Law_on_the_Control_of_Illicit_Trafficking_in_Narcotic_Drugs_Psychotropic_Substances_and_Precursors.pdf>
15. Bahrain. Law No. (15) of 2007 on Narcotic Drugs and Psychotropic Substances. Ministry of Health; 2007 [amended 2020]. Available from: <https://www.moh.gov.bh/NPP/NPPLaws?lang=en>
16. Bangladesh. Narcotics Control Act, 2018. Legislative and Parliamentary Affairs Division, Ministry of Law, Justice, and Parliamentary Affairs; 2018 Nov 14. Available from: <https://legislativediv.portal.gov.bd/sites/default/files/files/legislativediv.portal.gov.bd/page/5a6bca14_6a2e_44e4_b155_c8147d1edd65/27.%20Narcotics%20Control%20Act%2C%202018.pdf>
17. Barbados. Drug Abuse (Prevention and Control) Act, Cap. 131. Barbados Judicial System; 1990 [amended 2021]. Available from: <https://www.barbadoslawcourts.gov.bb/assets/content/pdfs/statutes/DrugAbuse%28PreventionandControl%29CAP131.pdf>
18. Belarus. Law on Narcotic Drugs, Psychotropic Substances, Their Precursors, and Analogues. National Legal Internet Portal of the Republic of Belarus; 2012 Jul 13. Available from: <https://cis-legislation.com/document.fwx?rgn=53188>
19. Belarus. Criminal Code of the Republic of Belarus, Article 328: Illicit Traffic in Narcotic Drugs, Psychotropic Substances, Their Precursors, and Analogues. National Legal Internet Portal of the Republic of Belarus; 1999 [amended 2019]. Available from: <https://legalizebelarus.org/en/press/article-328-of-the-criminal-code-of-republic-of-belarus/>
20. Belgium. Royal Decree of September 6, 2017, regulating narcotics and psychotropic substances in application of the law dated February 24, 1921. Federal Agency for Medicines and Health Products; 2017 Sep 6. Available from: <https://www.famhp.be/en/human_use/particular_products/specially_reglemented_substances/narcotics_psychotropics/legislation_substances>
21. Belize. Misuse of Drugs Act, Chapter 103. Belize Judiciary; 1990 [amended 2023]. Available from: <https://www.belizejudiciary.org/download/Laws-of-Belize-Update-2011/VOLUME%206A/Cap%20103%20Misuse%20of%20Drugs%20Act.pdf>
22. Benin. Law on the Control of Drugs and Precursors. 1997.
23. Bermuda. Misuse of Drugs Act 1972. Bermuda Laws Online; 1972 [amended 2017]. Available from: <https://www.bermudalaws.bm/Laws/Consolidated%20Law/1972/Misuse%20of%20Drugs%20Act%201972>
24. Bhutan. Narcotic Drugs, Psychotropic Substances and Substance Abuse Act of Bhutan 2015. Office of the Attorney General; 2015 May 11. Available from: <https://oag.gov.bt/wp-content/uploads/2010/05/Narcotic%20Drugs%20Psychotropic%20Substances%20and%20Substance%20Abuse%20Act%20of%20Bhutan%202015.pdf>
25. Bhutan. Narcotic Drugs, Psychotropic Substances and Substance Abuse (Amendment) Act of Bhutan 2018. Office of the Attorney General; 2018 Jan 8. Available from: <https://oag.gov.bt/wp-content/uploads/2024/07/Narcotic-Drugs-Psychotropic-Substance-and-Substance-Abuse-Amendment-Act-2018.pdf>
26. Bosnia and Herzegovina. National Strategy on Supervision over Narcotic Drugs, Prevention and Suppression of the Abuse of Narcotic Drugs in Bosnia and Herzegovina 2018–2023. Parliamentary Assembly of Bosnia and Herzegovina; 2018. Available from: <https://www.euda.europa.eu/system/files/publications/9424/National_drug_situation_report_Bosnia_and_Herzegovina.pdf>
27. Bosnia and Herzegovina. Law on Prevention and Suppression of Abuse of Narcotic Drugs. Official Gazette of Bosnia and Herzegovina; 2006.
28. Botswana. Illicit Traffic in Narcotic Drugs and Psychotropic Substances Act, 2018. Botswana Laws Online; 2018 Jun 29. Available from: <https://botswanalaws.com/consolidated-statutes/principle-legislation/illicit-traffic-in-narcotic-drugs-and-psychotropic-substances>
29. Botswana. Drugs and Related Substances Act, 1992. Botswana Laws Online; 1992 Sep 18. Available from: <https://botswanalaws.com/StatutesActpdf/1992Actpdf/DRUGS%20AND%20RELATED%20SUBSTANCES%20ACT%2C%2018%20OF%20192.pdf>
30. Brazil. Law No. 11,343 of August 23, 2006. Official Gazette of the Federative Republic of Brazil; 2006 Aug 23.
31. Brunei Darussalam. Misuse of Drugs Act, Chapter 27. Attorney General's Chambers; 1978 [revised 2013]. Available from: <https://www.agc.gov.bn/AGC%20Images/LAWS/ACT_PDF/cap027.pdf>
32. Bulgaria. Narcotic Substances and Precursors Control Act. State Gazette; 1999 Apr 2 [amended 2016 Jul 26].
33. Bulgaria. Penal Code, Articles 354a–354c. State Gazette; 1968 [amended 2017].
34. Burkina Faso. Law No. 017-99/AN of May 22, 1999, on the Control of Narcotic Drugs and Psychotropic Substances.
35. Burundi. Penal Code, Articles 505–513. Law No. 1/27 of December 29, 2017.
36. Burundi. Code of Criminal Procedure, Article 49. Law No. 1/09 of May 11, 2018.
37. Cambodia. Law on Drug Control. Phnom Penh: Royal Government of Cambodia; 2012 Jan 2. Available from: <https://sithi.org/medias/files/projects/sithi/law/Drug-Control-Law-2012-Final-English-Version.pdf>
38. Cameroon. Law No. 97/019 of August 7, 1997, on the Control of Narcotic Drugs, Psychotropic Substances, and Precursors, and on Extradition and Mutual Legal Assistance in Matters of Illicit Trafficking.
39. Canada. Controlled Drugs and Substances Act, S.C. 1996, c. 19. Department of Justice Canada; 1996. Available from: <https://laws-lois.justice.gc.ca/eng/acts/C-38.8/>
40. Canada. Cannabis Act, S.C. 2018, c. 16. Department of Justice Canada; 2018. Available from: <https://laws-lois.justice.gc.ca/eng/acts/C-24.5/>
41. Central African Republic. Penal Code.
42. Chad. Law No. 34/PR/95 of December 31, 1995, on the Control of Narcotic Drugs and Psychotropic Substances.
43. Chad. Law 006/PR/2018 on Combatting Trafficking in Persons.
44. Chile. Law No. 20.000, Establishing Sanctions for Illicit Trafficking of Narcotic Drugs and Psychotropic Substances. Official Gazette of the Republic of Chile; 2005 Feb 16.
45. China. Anti-Drug Law of the People's Republic of China. Beijing: National People's Congress; 2007 Dec 29. Available from: <https://english.court.gov.cn/2015-07/17/c_761568.htm>
46. China. Drug Administration Law of the People's Republic of China. Beijing: National Medical Products Administration; 2019 Aug 26. Available from: <https://english.nmpa.gov.cn/2019-09/26/c_773012.htm>
47. Colombia. Constitutional Court Ruling C-221 of 1994.
48. Union of the Comoros. Penal Code. Moroni: Government of the Union of the Comoros; 2021.
49. Republic of the Congo. Directorate of Pharmacy and Medicine.
50. Democratic Republic of the Congo. National Medicines Regulatory Authority.
51. Cook Islands. Narcotics and Misuse of Drugs Amendment Act 2023. Rarotonga: Parliament of the Cook Islands; 2023.
52. Costa Rica. Law No. 8204, Comprehensive Reform of the Law on Narcotic Drugs, Psychotropic Substances, Unauthorized Use Drugs, and Related Activities. San José: Legislative Assembly; 2001 Dec 17.
53. Croatia. Drug Abuse Prevention Act. Narodne novine; 2001 Nov.
54. Czech Republic. Criminal Code (Act No. 40/2009 Coll.).
55. Cuba. Penal Code. Havana: Government of the Republic of Cuba.
56. Cyprus. Narcotic Drugs and Psychotropic Substances Law of 1977. Law No. 29/1977. Nicosia: Government of Cyprus; 1977.
57. Cyprus. Narcotic Drugs and Psychotropic Substances (Amendment) Law of 2016. Law No. 57(I)/2016. Nicosia: Government of Cyprus; 2016.
58. Cyprus. Treatment of Convicted Users and Addicts Law of 2016. Law No. 41(I)/2016. Nicosia: Government of Cyprus; 2016.
59. Denmark. Euphoriant Substances Act. Copenhagen: Government of Denmark.
60. Denmark. Act No. 163 of 28 February 2012, amending the Euphoriant Substances Act. Copenhagen: Government of Denmark; 2012.
61. Denmark. Decree No. 1329 of 18 December 2008, amending the Decree on Narcotics No. 748. Copenhagen: Government of Denmark; 2008.
62. Denmark. Danish Medicines Act. Copenhagen: Government of Denmark.
63. Djibouti. Law No. 171/AN/81 on Psychotropic Substances. Djibouti City: Government of Djibouti; 1981.
64. Djibouti. Law No. 145/AN/91/2E on the Conditions of Practice of Pharmacy. Djibouti City: Government of Djibouti; 1991.
65. Djibouti. Penal Code, Chapter II. Djibouti City: Government of Djibouti.
66. Commonwealth of Dominica. Drugs (Prevention of Misuse) Act, Chapter 40:07. Roseau: Government of the Commonwealth of Dominica.
67. Dominican Republic. Law No. 50-88 on Drugs and Controlled Substances. Santo Domingo: Government of the Dominican Republic; 1988 May 30.
68. Dominican Republic. Law No. 17-95, Amending Law No. 50-88 on Drugs and Controlled Substances. Santo Domingo: Government of the Dominican Republic; 1995 Dec 17.
69. Dominican Republic. Decree No. 288-96, Establishing the Regulation of Law 50-88 on Drugs and Controlled Substances. Santo Domingo: Government of the Dominican Republic; 1996.
70. Democratic Republic of Timor-Leste. Law No. 10/2017 on Combating Drug Trafficking and Consumption. Dili: Government of Timor-Leste; 2017.
71. Ecuador. Law on Narcotic Drugs and Psychotropic Substances (Law 108). Quito: Government of Ecuador; 1990.
72. Arab Republic of Egypt. Law No. 182 of 1960 on the Control of Narcotic Substances. Cairo: Government of Egypt; 1960.
73. Arab Republic of Egypt. Decree No. 691 of 2014, Ministry of Health. Cairo: Government of Egypt; 2014.
74. Arab Republic of Egypt. Law No. 73 of 2021 Regulating the Appointment and Continuation in Public Office Positions. Cairo: Government of Egypt; 2021.
75. El Salvador. Law Regulating Drug-Related Activities (Decree No. 153 of 2004). San Salvador: Government of El Salvador; 2004.
76. Equatorial Guinea. Criminal Code. Malabo: Government of Equatorial Guinea.
77. Eritrea. Proclamation No. 36/1993: A Proclamation to Provide for the Regulation of Pharmaceuticals, Medical Equipment, and Cosmetics. Asmara: Government of Eritrea; 1993.
78. Eritrea. Penal Code of the State of Eritrea. Asmara: Government of Eritrea; 2015
79. Estonia. Act on Narcotic Drugs and Psychotropic Substances and Precursors Thereof. Tallinn: Government of Estonia; 1997.
80. Estonia. Regulation No. 73 of 2005 on Handling of Narcotic Drugs and Psychotropic Substances. Tallinn: Ministry of Social Affairs; 2005.
81. Ethiopia. Penal Code of 1956. Addis Ababa: Government of Ethiopia; 1956.
82. Ethiopia. Pharmacy Regulation of 1964. Addis Ababa: Government of Ethiopia; 1964.
83. Fiji. Illicit Drugs Control Act 2004. Suva: Government of Fiji; 2004.
84. Finland. Narcotics Act (373/2008). Helsinki: Government of Finland; 2008.
85. Finland. Government Decree on Psychoactive Substances Banned from the Consumer Market (1130/2014). Helsinki: Government of Finland; 2014.
86. Finland. Government Decree on Substances, Preparations, and Plants Considered as Narcotics (543/2008). Helsinki: Government of Finland; 2008.
87. France. Law No. 70-1320 of December 31, 1970, on Measures Relating to the Fight Against Drug Abuse and the Suppression of Trafficking and Use of Toxic Substances. Paris: Government of France; 1970.
88. France. Decree of February 22, 1990, Establishing the List of Substances Classified as Narcotics and Psychotropic Substances. Paris: Government of France; 1990.
89. Gabon. Penal Code Act. Libreville: Government of Gabon; 2018.
90. Georgia. Law on Narcotic Drugs, Psychotropic Substances and Precursors, and Narcological Assistance. Tbilisi: Parliament of Georgia; 2012. Available from: <https://matsne.gov.ge/en/document/view/1670322?publication=18>
91. Germany. Narcotic Drugs Act (Betäubungsmittelgesetz, BtMG). Available from: <https://www.bundesgesundheitsministerium.de/fileadmin/Dateien/3_Downloads/Gesetze_und_Verordnungen/GuV/N/Narcotic_Drugs_18_12_2009.pdf>
92. Germany. New Psychoactive Substances Act (NpSG). Available from: <https://www.gesetze-im-internet.de/npsg/>
93. Ghana. Narcotic Drugs (Control, Enforcement and Sanctions) Law, 1990 (PNDCL 236). Available from: <https://new-ndpc-static1.s3.amazonaws.com/CACHES/PUBLICATIONS/2016/09/04/NARCOTIC%2BDRUGS.pdf>
94. Ghana. Narcotics Control Commission Act, 2020 (Act 1019). Available from: <https://www.ncc.gov.gh/history/>
95. Greece. Law 3459/2006 – Code of Laws for Drugs. Available from: <https://www.unodc.org/LSS/Country/DetailsLegalSystem?code=DLIL&country=GR>
96. Denmark. Danish Penal Code. Available from: <https://www.ojp.gov/ncjrs/virtual-library/abstracts/greenland-criminal-code>
97. Grenada. Drug Abuse (Prevention and Control) Act, 1992. Available from: <https://grenadaparliament.gd/wp-content/uploads/2021/08/Cap84A-DRUG-ABUSE-PREVENTION-AND-CONTROL-ACT.pdf>
98. Guam. Guam Uniform Controlled Substances Act, Chapter 67, Title 9, Guam Code Annotated. Available from: <https://guamcourts.org/CompilerofLaws/GCA/09gca/9gc067.PDF>
99. Guam. Guam Opioid Overdose Prevention Act of 2023, Chapter 16, Title 10, Guam Code Annotated. Available from: <https://www.guamcourts.org/CompilerofLaws/GCA/10gca/10gc016.PDF>
100. Guam. Guam Cannabis Industry Act, Chapter 8, Title 11, Guam Code Annotated. Available from: <https://guamcourts.org/CompilerofLaws/GCA/11gca/11gc008.PDF>
101. Guatemala. Law Against Drug Activity, Decree No. 48-92. 1992. Available from: <https://importlicensing.wto.org/sites/default/files/members/59/Decreto%20No.48-92%20-%20Narcotr%C3%A1fico_24.09.1992.pdf>
102. Guatemala. Law Against Organized Crime, Decree No. 21-2006. 2006. Available from: <https://mingob.gob.gt/wp-content/uploads/2020/10/10_LeyContraDelincuenciaOrganizada.pdf>
103. Guinea. Law L/94/005/CTRN on the Control of Narcotic Drugs and Psychotropic Substances. 1994.
104. Guinea-Bissau. Law on the Control of Narcotic Drugs and Psychotropic Substances.
105. Guyana. Narcotic Drugs and Psychotropic Substances (Control) (Amendment) Act. 2021. Available from: <https://mpag.gov.gy/wp-content/uploads/2022/03/2-Bill-No.-12-of-2021.pdf>
106. Haiti. Law on the Control of Narcotic Drugs and Psychotropic Substances.
107. Honduras. Law on the Improper Use and Illicit Trafficking of Drugs and Psychotropic Substances, Decree No. 126-89. 1989
108. Honduras. Amendment to the Law on the Improper Use and Illicit Trafficking of Drugs and Psychotropic Substances, Decree No. 86-93. 1993.
109. Hungary. Act C of 2012 on the Criminal Code. 2012. Available from: <https://eucpn.org/document/hungarian-policy-on-drugs-0>
110. Hungary. Act XCV of 2005 on Medicinal Products for Human Use and on the Amendment of Other Laws Regulating the Pharmaceutical Market. 2005. Available from: <https://drogfokuszpont.hu/wp-content/uploads/regulation_NPS_HU.pdf>
111. Hungary. Government Decree 66/2012 (IV. 2.) on activities related to narcotic drugs, psychotropic substances, and new psychoactive substances, on scheduling of these substances, and on modification of schedules. 2012. Available from: <https://drogfokuszpont.hu/wp-content/uploads/regulation_NPS_HU.pdf>
112. Iceland. Act No. 65/1974 on Narcotic Drugs. 1974. Available from: <https://importlicensing.wto.org/content/act-no651974-narcotic-drugs>
113. Iceland. Medicinal Products Act No. 100/2020. 2020. Available from: <https://www.government.is/library/01-Ministries/Ministry-of-HealTh/PDF-skjol/Lyfjal%C3%B6g%20nr.%20100.2020%20-%20ensk%20%C3%BE%C3%BD%C3%B0ing.pdf>
114. Iceland. Regulation No. 233/2001 on Habit-Forming and Narcotic Substances and Other Controlled Substances. 2001. Available from: <https://www.ima.is/regulated_entities/narcotic-drugs/>
115. Iceland. Regulation No. 212/1998 on Importation by Individuals of Medicinal Products for Personal Use. 1998. Available from: <https://www.ima.is/licences/importation-by-individuals-of-medicinal-products-for-personal-use/>
116. India. Narcotic Drugs and Psychotropic Substances Act, 1985. Available from: <https://www.indiacode.nic.in/bitstream/123456789/1791/1/A1985-61.pdf>
117. India. Prevention of Illicit Traffic in Narcotic Drugs and Psychotropic Substances Act, 1988. Available from: <https://www.indiacode.nic.in/bitstream/123456789/15400/1/pitndpsact1988.pdf>
118. Indonesia. Ministry of Health Regulation No. 5 of 2020 amending Law No. 35 of 2009 on Narcotics. 2020.
119. Indonesia. Law No. 5 of 1997 on Psychotropic Substances. 1997.
120. Iraq. Law No. 50 of 2017 on Narcotic Drugs and Psychotropic Substances. 2017.
121. Ireland. Misuse of Drugs Regulations 2017. 2017. Available from: <https://www.irishstatutebook.ie/eli/2017/si/173/made/en/print>
122. Ireland. Misuse of Drugs (Amendment) Regulations 2017. 2017. Available from: <https://www.irishstatutebook.ie/eli/2017/si/532/made/en/print>
123. Israel. Dangerous Drugs Ordinance [New Version], 5733-1973. 1973.
124. Israel. Drug Control Authority Law, 5748-1988. 1988.
125. Italy. Presidential Decree No. 309 of October 9, 1990, Consolidation of the laws governing drugs and psychotropic substances, the prevention, treatment, and rehabilitation of drug addicts. 1990. Available from: <https://www.iss.it/documents/20126/0/ANN_20_01_12.pdf>
126. Italy. Law No. 49 of February 21, 2006, Conversion into law, with amendments, of Decree-Law No. 272 of December 30, 2005, concerning urgent measures to guarantee the safety and financing of the next Winter Olympic Games, as well as the functioning of the administration of the interior. 2006.
127. Italy. Law No. 79 of May 16, 2014, Conversion into law, with amendments, of Decree-Law No. 36 of March 20, 2014, concerning urgent provisions on drug substances and psychotropic substances, as well as on the therapeutic use of cannabis-based medicines. 2014.
128. Italy. Decree No. 146 of June 16, 2010, Amending the list of controlled substances contained in Decree 309/1990 "Consolidation of the laws governing drugs and psychotropic substances, the prevention, treatment, and rehabilitation of drug addicts." 2010.
129. Jamaica. The Dangerous Drugs (Amendment) Act. 2021. Available from: <https://laws.moj.gov.jm/library/act-of-parliament/3-of-2021-the-dangerous-drugs-amendment-act>
130. Japan. Narcotics and Psychotropics Control Act. Act No. 14 of 1953. Available from: <https://www.japaneselawtranslation.go.jp/en/laws/view/2849>
131. Japan. Cannabis Control Act. Act No. 124 of 1948.
132. Japan. Stimulants Control Act. Act No. 252 of 1951.
133. Jordan. Narcotic Drugs and Psychotropic Substances Law No. 23 of 2016. 2016.
134. Jordan. Amendments to the Narcotic Drugs and Psychotropic Substances Law. 2021.
135. Kazakhstan. Law No. 279-I of July 10, 1998, On Narcotic Drugs, Psychotropic Substances, Their Analogues and Precursors and Measures to Combat Their Illicit Trafficking and Abuse. 1998. Available from: <https://adilet.zan.kz/eng/docs/Z980000279_>
136. Kazakhstan. Government Decree No. 470 of July 3, 2019, On Approval of the List of Narcotic Drugs, Psychotropic Substances and Precursors to be Controlled in the Republic of Kazakhstan. 2019. Available from: <https://adilet.zan.kz/eng/docs/P1900000470>
137. Kazakhstan. Government Decree No. 366 of June 5, 2019, On Approval of the Rules for Formation of the List of Narcotic Drugs, Psychotropic Substances and Precursors to be Controlled in the Republic of Kazakhstan. 2019. Available from: <https://adilet.zan.kz/eng/docs/P1900000366>
138. Kenya. Narcotics, Drugs and Psychotropic Substances (Control) (Amendment) Act, 2022. Act No. 4 of 2022. Available from: <https://new.kenyalaw.org/akn/ke/act/1994/4>
139. Kiribati. Dangerous Drugs Ordinance. Cap. 30. Available from: <http://www.paclii.org/ki/legis/consol_act/ddo233/>
140. Kiribati. Pharmacy and Poisons Ordinance. Cap. 31.
141. Kuwait. Law No. 74 of 1983, Concerning the Control of Narcotic Drugs and Psychotropic Substances. 1983.
142. Kuwait. Law No. 48 of 1987, Amending Certain Provisions of Law No. 74 of 1983 on Narcotic Drugs and Psychotropic Substances. 1987.
143. Kyrgyzstan. Law on Narcotic Drugs, Psychotropic Substances, Their Analogues, and Precursors. 2024.
144. Laos. Law on Narcotic Drugs and Psychotropic Substances. 2007.
145. Laos. Penal Code. 2017.
146. Laos. Ministry of Health. Decision No. 3789/MOH on the Control of Hemp for Medication and Products. 2022.
147. Latvia. Law on Procedures for the Legal Trade of Narcotic and Psychotropic Substances and Medicinal Products. 1996. Available from: <https://likumi.lv/ta/en/en/id/40283-on-procedures-for-the-legal-trade-of-narcotic-and-psychotropic-substances-and-medicinal-products>
148. Latvia. Cabinet Regulation No. 847, Regulations Regarding Narcotic Substances, Psychotropic Substances and Precursors to be Controlled in Latvia. 2005.
149. Latvia. National Programme on Drug Control and Drug Addiction Restriction for 2011–2017. 2011.
150. Lebanon. Law No. 673 on Narcotic Drugs, Psychotropic Substances, and Precursors. 1998 Mar 16.
151. Lebanon. Inter-Ministerial Substance Use Response Strategy for Lebanon 2016–2021. Available from: <https://www.moph.gov.lb/userfiles/files/Inter-minsiterial%20Substance%20Use%20Response%20Strategy%20for%20Lebanon%202016-2021-English.pdf>
152. Lesotho. Drugs of Abuse (Amendment) Act, 2022. Act No. 1 of 2022. Available from: <https://gazettes.africa/archive/ls/2022/ls-government-gazette-dated-2022-01-27-no-4.pdf>
153. Liberia. Controlled Drugs and Substances Act. 2014.
154. Liberia. Liberia Drug Enforcement Agency Act. 2014.
155. Liberia. Medicines & Health Products Regulatory Authority Act. 2010.
156. Libya. Law No. (7) of 1990 on Narcotic Drugs and Psychotropic Substances. 1990.
157. Libya. Decree No. (142) of 2012 on Dissolving the Anti-Narcotic Drugs and Psychotropic Substances Agency. 2012. Available from: <https://security-legislation.ly/latest-laws/decree-no-142-of-2012-on-dissolving-the-anti-narcotic-drugs-and-psychotropic-substances-agency/>
158. Republic of Lithuania. Law on the Control of Narcotic Drugs and Psychotropic Substances. 1998 Jan 8. Available from: <https://e-seimas.lrs.lt/portal/legalAct/lt/TAD/TAIS.315268>
159. Republic of Lithuania. Criminal Code of the Republic of Lithuania. Available from: <https://www.e-tar.lt/portal/lt/legalAct/TAR.2B866DFF7D43/asr>
160. Republic of Lithuania. Law on the Control of Precursors of Narcotic Drugs and Psychotropic Substances. 1999 Jun 1. Available from: <https://e-seimas.lrs.lt/rs/legalact/TAD/TAIS.209581/>
161. Luxembourg. Grand Ducal Regulations on Controlled Substances. Available from: <https://sante.public.lu/dam-assets/fr/publications/e/etat-drogue-gdl-rapport-relis-2020-fr-en/etat-drogue-gdl-rapport-relis-2020-en.pdf>
162. Luxembourg. Law of 17 March 1992 Approving the United Nations Convention Against Illicit Traffic in Narcotic Drugs and Psychotropic Substances. 1992 Mar 17.
163. Luxembourg. Amended Law of 19 February 1973 on the Sale of Medicinal Substances and the Fight Against Drug Addiction. 1973 Feb 19.
164. Republic of Madagascar. Law No. 97-039 on the Control of Narcotic Drugs, Psychotropic Substances, and Precursors. 1997 Nov 4.
165. Malawi. Pharmacy, Medicines and Poisons Act. 1988 May 18. Available from: <https://malawilii.org/akn/mw/act/1988/15/eng%402014-12-31>
166. Malawi. Dangerous Drugs Act. 1956 Apr 1. Available from: <https://malawilii.org/akn/mw/act/1955/28/eng%402014-12-31>
167. Malaysia. Dangerous Drugs (Amendment) Act 2017. Act A1548. 2017. Available from: <https://lom.agc.gov.my/ilims/upload/portal/akta/outputaktap/1709273_BI/Act%20234%20Final%201.11.2021.pdf>
168. Malaysia. Control of Drugs and Cosmetics Regulations 1984. P.U.(A) 223/84. 1984. Available from: <https://pharmacy.moh.gov.my/sites/default/files/document-upload/control-drugs-and-cosmetics-regulation-1984.pdf>
169. Malaysia. Drug Dependants (Treatment and Rehabilitation) Act 1983. Act 283. 1983.
170. Republic of Maldives. Drugs Act. Law No. 17/2011. 2011. Available from: <https://www.issup.net/files/2020-08/2020%20Drugs%20Act%20of%20the%20Maldives.pdf>
171. Republic of Mali. Law No. 01-078 on the Control of Drugs and Precursors. 2001 Jul 18.
172. Malta. Drug Dependence (Treatment not Imprisonment) Act. Cap. 537. 2014.
173. Malta. Dangerous Drugs Ordinance. Cap. 101. 1939.
174. Republic of the Marshall Islands. Narcotic Drugs (Prohibition and Control) Act 1987. 7 MIRC Ch. 9. 1987 Mar 20.
175. Republic of the Marshall Islands. Prohibited Drugs Prohibition and Control (Amendment) Act 2020. P.L. 2020-12. 2020 Dec 23.
176. Islamic Republic of Mauritania. Law No. 93-37 on the Suppression of the Production, Trafficking, and Illicit Use of Narcotic Drugs and Psychotropic Substances. 1993 Jul 20.
177. Islamic Republic of Mauritania. Decree No. 90-170 Establishing a National Commission to Combat Drugs and Psychotropic Substances. 1990 Nov 13.
178. Mauritius. Dangerous Drugs Act 2000. Act No. 41 of 2000. 2000 Dec 29. Available from: <https://health.govmu.org/health/wp-content/uploads/2023/03/DANGEROUS-DRUGS-ACT-2000-1.pdf>
179. Mauritius. Pharmacy Act 1983. Act No. 60 of 1983. 1983 Dec 30.
180. Mexico. Ley General de Salud. Diario Oficial de la Federación. 1984 Feb 7.
181. Mexico. Código Penal Federal. Diario Oficial de la Federación. 1931 Aug 14.
182. Mexico. Código Nacional de Procedimientos Penales. Diario Oficial de la Federación. 2014 Mar 5.
183. Republic of Moldova. Law No. 382-XIV on the Circulation of Narcotic Drugs, Psychotropic Substances, and Precursors. 1999 May 6.
184. Republic of Moldova. Law No. 1409-XIII on Drugs. 1997 Dec 17.
185. Mongolia. Law on Narcotic Drugs and Psychotropic Substances. 2002.
186. Mongolia. Drugs Act. 1998.
187. Mongolia. Law on Medicines and Medical Devices. 2020. Available from: <https://legalinfo.mn/en/edtl/16760184774901>
188. Montenegro. Law on the Prevention of Drug Abuse. Official Gazette of Montenegro. Nos. 28/2011 and 35/2013.
189. Montenegro. Law on the Control of Production and Trade of Substances That Can Be Used in the Production of Narcotic Drugs and Psychotropic Substances. Official Gazette of Montenegro. No. 83/09.
190. Montenegro. Law on Medicines. Official Gazette of Montenegro. No. 80/20.
191. Montenegro. Regulation Determining the List of Drugs, Psychotropic Substances, and Plants That Can Be Used for the Production of Drugs.
192. Kingdom of Morocco. Law No. 1-73-282 on Measures to Combat Drug Addiction. 1974 Aug 21.
193. Kingdom of Morocco. Law No. 13.10 amending the Penal Code. 2011 Jan 24.
194. Kingdom of Morocco. Law No. 21-81 on the Prohibition of Cultivation of Narcotic Plants. 1981 Nov 25.
195. Republic of Mozambique. Law No. 3/97 on the illicit production, trafficking, and consumption of narcotic drugs, psychotropic substances, precursors, and other substances with similar effects. 1997 Mar 13.
196. Myanmar. The Narcotic Drugs and Psychotropic Substances (Amendment) Law. 2018 Feb 14. Available from: <https://www.burmalibrary.org/en/pyidaungsu-hluttaw-law-no-62018-drugs-and-psychotropic-drugs-amendment-law>
197. Namibia. Medicines and Related Substances Control Amendment Act, 2007. Act No. 8 of 2007. Available from: <https://namiblii.org/akn/na/act/2007/8/eng%402007-12-28>
198. Namibia. Abuse of Dependence-Producing Substances and Rehabilitation Centres Act, 1971. Act No. 41 of 1971.
199. Nepal. Narcotic Drugs (Control) Act, 2033 (1976). Available from: <https://www.dda.gov.np/download/narcotic-drugs-control-act-2033-1976.pdf>
200. Netherlands. Opium Act. 1919. Available from: <https://wetten.overheid.nl/BWBR0001941/2020-11-17>
201. New Zealand. Drug and Substance Checking Legislation Act 2021. Available from: <https://www.legislation.govt.nz/act/public/2021/0050/latest/whole.html>
202. New Zealand. Misuse of Drugs Regulations 1977. Available from: <https://www.health.govt.nz/regulation-legislation/medicines-control/controlled-drugs>
203. New Zealand. Misuse of Drugs Act 1975. Available from: <https://www.legislation.govt.nz/act/public/1975/0116/latest/whole.html>
204. Republic of Nicaragua. Ley de Reforma y Adiciones a la Ley No. 177, Ley de Estupefacientes, Sicotrópicos y Sustancias Controladas. Law No. 285. 1999 Apr 16
205. Republic of Nicaragua. Reglamento de la Ley No. 292, Ley de Medicamentos y Farmacias. Decree No. 6-99. 1999.
206. Republic of Nicaragua. Ley de Prevención, Investigación y Persecución del Crimen Organizado y de la Administración de los Bienes Incautados, Decomisados y Abandonados. Law No. 735. 2010.
207. Republic of Niger. Ordinance No. 99-42 of September 23, 1999, relating to the fight against drugs in Niger. JORN. 1999 Dec 1;(23):1003.
208. Republic of Niger. Order No. 459/MSP/DGSP/DPHL/MT of December 17, 2013, listing Tramadol in Group A of Table II of Ordinance No. 99-42. JORN. 2013 Dec 17.
209. Republic of Niger. Decree No. 2015-295/PRN/MISPD/ACR of June 5, 2015, determining the missions, organization, and functioning of the Central Office for the Repression of Illicit Drug Trafficking (OCRTIS). JORN. 2015 Jun 5.
210. Nigeria. Controlled Medicines Regulations 2021. Available from: <https://www.nafdac.gov.ng/wp-content/uploads/Files/Resources/Regulations/REGULATIONS_2021/CONTROLLED-MEDICINES-REGULATIONS-2021.pdf>
211. Democratic People's Republic of Korea. Narcotics Control Law. 2005 May 17.
212. Democratic People's Republic of Korea. Law on the Prevention of Drug-Related Crimes. 2021 Jul 1.
213. Norway. Regulations on Narcotic Drugs. FOR-2013-02-14-199. Available from: <https://www.lovdata.no/dokument/SF/forskrift/2013-02-14-199>
214. Sultanate of Oman. Royal Decree No. 17/99 Promulgating the Law on Combating Narcotic Drugs and Psychotropic Substances. 1999 Mar 6. Available from: <https://decree.om/1999/rd19990017/>
215. Sultanate of Oman. Royal Decree No. 34/2015 Amending the Law on Combating Narcotic Drugs and Psychotropic Substances. 2015 Oct 5.
216. Pakistan. Control of Narcotic Substances Act, 1997. Act No. XXV of 1997. Available from: <https://pakistancode.gov.pk/pdffiles/administrator739c7aa745c5afab5decf2e100caf1c5.pdf>
217. Pakistan. Anti-Narcotics Force Act, 1997. Act No. III of 1997. Available from: <https://pakistancode.gov.pk/pdffiles/administrator7db4367e97eddbb09333e0a9f87d4dfc.pdf>
218. Pakistan. Control of Narcotic Substances (Amendment) Act, 2022. Available from: <https://www.president.gov.pk/news/president-assents-to-control-of-narcotic-substances-amendment-bill-2022>
219. Republic of Panama. Law No. 419 on Controlled Drugs. 2022.
220. Republic of Panama. Law No. 23 on the Control of Narcotic Drugs. 1986.
221. Papua New Guinea. Dangerous Drugs (Amendment) Act 2021. 2021 Nov 25.
222. Papua New Guinea. Controlled Substances Act 2021. 2021 Dec 3.
223. Paraguay. Ley No. 1.340/88 sobre Tráfico de Estupefacientes y Sustancias Peligrosas. 1988.
224. Paraguay. Ley No. 1.015/96 que Previene y Reprime el Lavado de Dinero o Bienes. 1996.
225. Paraguay. Ley No. 1.016/97 que Regula Sustancias Químicas y Precursores. 1997.
226. Peru. Decreto Legislativo No. 122 que establece medidas de control sobre drogas narcóticas y sustancias psicotrópicas. 1981.
227. Peru. Ley No. 28002 que regula el uso lícito de la hoja de coca. 2003.
228. Peru. Decreto Legislativo No. 1241 que fortalece la lucha contra el tráfico ilícito de drogas. 2015.
229. Philippines. Republic Act No. 9165: Comprehensive Dangerous Drugs Act of 2002. 2002 Jun 7. Available from: <https://lawphil.net/statutes/repacts/ra2002/ra_9165_2002.html>
230. Philippines. Dangerous Drugs Board Regulation No. 3, Series of 2003. 2003. Available from: <https://ddb.gov.ph/images/Board_Regulation/2003/Bd.%20Reg.%203%2003.pdf>
231. Philippines. Dangerous Drugs Board. Updated Lists of Scheduled Controlled Substances as of 25 April 2022. 2022 Apr 25. Available from: <https://ddb.gov.ph/wp-content/uploads/2022/04/Updated-Lists-of-controlled-substances-as-of-25-April-2022.pdf>
232. Poland. Regulation of the Minister of Health of 16 March 2017 on Detailed Conditions and Mode of Issuing Approvals and Documents Necessary for Import, Export, Intra-Community Acquisition or Intra-Community Supply of Narcotic Drugs, Psychotropic Substances, or Category 1 Precursors. Available from: <https://www.gov.pl/web/chief-pharmaceutical-inspectorate/controlled-substances/>
233. Poland. Regulation on the List of Psychotropic Substances, Narcotic Drugs, and New Psychoactive Substances.
234. Poland. Act of 29 July 2005 on Counteracting Drug Addiction
235. Portugal. Decreto-Lei n.º 15/93 de 22 de janeiro. Diário da República. 1993 Jan 22. Available from: <https://dre.pt/dre/detalhe/decreto-lei/15-1993-344933>
236. Portugal. Lei n.º 30/2000 de 29 de novembro. Diário da República. 2000 Nov 29. Available from: <https://dre.pt/dre/detalhe/lei/30-2000-452407>
237. Puerto Rico. Controlled Substances Act of Puerto Rico. Act No. 4 of June 23, 1971.
238. Puerto Rico. Medical Cannabis Act. Act No. 42 of July 9, 2017.
239. Qatar. Law No. 9 of 1987 on the Control and Regulation of Narcotic Drugs and Dangerous Psychotropic Substances.
240. Romania. Law No. 143 of July 26, 2000, on Preventing and Combating Illicit Drug Trafficking and Use.
241. Romania. Law No. 339 of December 29, 2005, on the Legal Regime of Plants, Substances, and Preparations with Narcotic and Psychotropic Effects.
242. Romania. Law No. 194 of November 10, 2011, on Combating Operations with Products Susceptible to Having Psychoactive Effects, Other Than Those Provided by Current Regulations.
243. Russian Federation. Federal Law No. 3-FZ of January 8, 1998, "On Narcotic Drugs and Psychotropic Substances."
244. Russian Federation. Government Decree No. 681 of June 30, 1998.
245. Russian Federation. Federal Law No. 323-FZ of November 21, 2011, "On the Fundamentals of Health Protection of Citizens in the Russian Federation."
246. Rwanda. Law No. 03/2012 of 15 February 2012 Governing Narcotic Drugs, Psychotropic Substances, and Precursors in Rwanda. Available from: <https://rwandalii.org/akn/rw/act/law/2012/3>
247. Rwanda. Ministerial Order Establishing the List of Narcotic Drugs and Their Precursors. Available from: <https://rwandalii.org/akn/rw/act/mo/minister-of-health/2019/1/eng@2019-03-11/source>
248. Saint Kitts and Nevis. Drugs (Prevention & Abatement of the Misuse and Abuse of Drugs) Act, Cap 9.08. Available from: <https://parliament.gov.kn/wp-content/uploads/2017/06/drugs_act.pdf>
249. Saint Kitts and Nevis. Amendments to the Drugs (Prevention & Abatement of the Misuse and Abuse of Drugs) Act, 2019.
250. Saint Lucia. Drugs (Prevention of Misuse) Act.
251. Saint Lucia. Amendments to the Drugs (Prevention of Misuse) Act, 2021.
252. Saint Vincent and the Grenadines. Drugs (Prevention of Misuse) Act. Available from: <https://assembly.gov.vc/assembly/index.php/acts-sp-1308988606>
253. Saint Vincent and the Grenadines. Drugs (Prevention of Misuse) (Amendment) Act, 2019. Available from: <https://pmoffice.gov.vc/pmoffice/images/stories/PDF/Drugs-Prevention-of-Misuse-Amendment-Bill-2018KDeditsAlternative.pdf>
254. Saint Vincent and the Grenadines. Medicinal Cannabis Industry Act, 2018. Available from: <https://mca.vc/legislation/saint-vincent-and-the-grenadines-medicinal-cannabis-industry-act-2018/>
255. Saint Vincent and the Grenadines. Cannabis Cultivation (Amnesty) Act, 2018. Available from: <https://mca.vc/legislation/>
256. Samoa. Narcotics Act 1967. Available from: <https://www.paclii.org/ws/legis/consol_act_2019/na1967114.pdf>
257. São Tomé and Príncipe. Penal Code.
258. Saudi Arabia. Law of Combating Narcotics and Psychotropic Substances. Available from: <https://www.sfda.gov.sa/sites/default/files/2021-11/NarcoticDrugsPsychotropicSubstances.pdf>
259. Saudi Food and Drug Authority. Procedures and Controls for Narcotic Drugs and Psychotropic Substances. Available from: <https://www.sfda.gov.sa/en/regulations/85958>
260. Senegal. Law No. 97-18 of 1 December 1997 on the Control of Narcotic Drugs, Psychotropic Substances, and Precursors.
261. Senegal. Amendment to Law No. 97-18 on the Control of Narcotic Drugs, Psychotropic Substances, and Precursors. 2006.
262. Serbia. Law on Psychoactive Controlled Substances. Official Gazette of the Republic of Serbia, No. 99/2010 and 57/2018.
263. Serbia. Law on Substances Used in Illicit Manufacturing of Narcotic Drugs and Psychotropic Substances. Official Gazette of the Republic of Serbia, No. 107/2005 and 25/2019.
264. Serbia. Criminal Code. Official Gazette of the Republic of Serbia, No. 111/2009.
265. Seychelles. Misuse of Drugs Act, 2016. Available from: <https://seylii.org/akn/sc/act/2016/5/eng%402016-04-20>
266. Seychelles. Misuse of Drugs (Amendment) Act, 2017. Available from: <https://seylii.org/akn/sc/act/2017/14/eng%402017-08-17>
267. Seychelles. Misuse of Drugs (Cannabidiol-based Products for Medical Purposes) Regulations, 2020. Available from: <https://www.gazette.sc/sites/default/files/2020-12/SI%2025%202020%20-%20Misuse%20of%20Drugs%20Act%202020.pdf>
268. Sierra Leone. National Drugs Control Act, 2008. Available from: <https://www.sierra-leone.org/Laws/2008-10.pdf>
269. Sierra Leone. National Drugs Control (Amendment) Act, 2008. Available from: <https://www.sierra-leone.org/Laws/2008-13.pdf>
270. Singapore. Misuse of Drugs Act 1973. Available from: <https://sso.agc.gov.sg/Act/MDA1973>
271. Singapore. Misuse of Drugs (Amendment) Act 2023. Available from: <https://sso.agc.gov.sg/Acts-Supp/12-2023/Published/20230424>
272. Slovakia. Act No. 139/1998 Coll. on Narcotic Substances, Psychotropic Substances, and Preparations. Available from: <https://www.slov-lex.sk/pravne-predpisy/SK/ZZ/1998/139/>
273. Slovakia. Act No. 300/2005 Coll. Criminal Code. Available from: <https://www.slov-lex.sk/pravne-predpisy/SK/ZZ/2005/300/>
274. Slovakia. Act No. 372/1990 Coll. on Offenses. Available from: <https://www.slov-lex.sk/pravne-predpisy/SK/ZZ/1990/372/>
275. Slovenia. Production and Trade in Illicit Drugs Act. Official Gazette of the Republic of Slovenia, No. 108/1999.
276. Slovenia. Act Regulating the Prevention of the Use of Illicit Drugs and the Treatment of Drug Users. Official Gazette of the Republic of Slovenia, No. 98/1999.
277. Slovenia. Criminal Code. Official Gazette of the Republic of Slovenia, No. 50/2012.
278. Solomon Islands. Dangerous Drugs Act (Cap. 98).
279. Solomon Islands. Penalties Miscellaneous Amendments Act 2009. Available from: <https://www.parliament.gov.sb/sites/default/files/legislation/Acts/Penalties%20Miscellaneous%20%28Amendments%29%20Act%202009.pdf>
280. Somalia. Law No. 46: Regulation of Production, Trade, and Distribution of Medical Drugs. 1970 Mar 3.
281. Somalia. National Medicine Regulatory Authority. Ministry of Health Somalia. Available from: <https://moh.gov.so/en/nmra/>
282. South Africa. Drugs and Drug Trafficking Act 140 of 1992. Available from: <https://www.gov.za/documents/drugs-and-drug-trafficking-act>
283. South Africa. Prevention of and Treatment for Substance Abuse Act 70 of 2008. Available from: <https://www.gov.za/documents/prevention-and-treatment-substance-abuse-act>
284. South Africa. Medicines and Related Substances Act 101 of 1965. Available from: <https://www.gov.za/documents/medicines-and-related-substances-act>
285. Republic of Korea. Narcotics Control Act. Available from: <https://elaw.klri.re.kr/eng_service/lawView.do?hseq=37716&lang=ENG>
286. Republic of Korea. Enforcement Rule of the Narcotics Control Act. Available from: <https://www.mfds.go.kr/eng/brd/m_18/down.do?brd_id=eng0003&data_tp=A&file_seq=1&seq=71536>
287. Republic of South Sudan. Drug and Food Control Authority Act, 2012. Act No. 37.
288. Spain. Law 17/1967 of 8 April on Narcotic Drugs.
289. Spain. Royal Decree 2829/1977 of 6 October, regulating the manufacture, distribution, and prescription of psychotropic substances and preparations.
290. Spain. Order of 14 January 1981 on Psychotropic Substances.
291. Spain. Royal Decree 1675/2012 of 14 December, regulating official prescriptions and special requirements for prescribing and dispensing narcotic drugs.
292. Spain. Organic Law 4/2015 of 30 March on the Protection of Citizen Security.
293. Sri Lanka. Poisons, Opium, and Dangerous Drugs Ordinance, No. 17 of 1929. Available from: <https://www.nddcb.gov.lk/Docs/acts/25345.pdf>
294. Sri Lanka. Conventions Against Illicit Traffic in Narcotic Drugs and Psychotropic Substances Act, No. 1 of 2008.
295. Sri Lanka. Drug Dependent Persons (Treatment and Rehabilitation) Act, No. 54 of 2007. Available from: <https://www.nddcb.gov.lk/policy-regulation-documents.php>
296. Sri Lanka. National Policy for the Prevention and Control of Drug Abuse. Available from: <https://www.nddcb.gov.lk/policy-regulation-documents.php>
297. Sudan. Narcotic Drugs and Psychotropic Substances Act, 1994
298. Suriname. Act of 12 February 1998 concerning confirmation of the Act on Narcotic Drugs.
299. Sweden. Narcotic Drugs (Punishments) Act, 1968:64. Available from: <https://www.ojp.gov/ncjrs/virtual-library/abstracts/narcotic-drugs-punishments-act-current-swedish-legislation-narcotic>
300. Sweden. Act on the Control of Narcotic Drugs, 1992:860. Available from: <https://www.ojp.gov/ncjrs/virtual-library/abstracts/act-control-narcotic-drugs-current-swedish-legislation-narcotic>
301. Sweden. Ordinance on the Control of Narcotic Drugs, 1993:858. Available from: <https://www.ojp.gov/ncjrs/virtual-library/abstracts/ordinance-control-narcotic-drugs-current-swedish-legislation>
302. Switzerland. Federal Act on Narcotics and Psychotropic Substances (Narcotics Act, NarcA).
303. Switzerland. Ordinance of the Swiss Agency for Therapeutic Products on the Control of Narcotics and Psychotropic Substances (Betäubungsmittelverordnung Swissmedic, BetmV-Swissmedic).
304. Switzerland. Ordinance on Narcotics Control (Narcotics Control Ordinance, NarcCO)
305. Syrian Arab Republic. Law No. 2 of 1993 (Drug Law).
306. Taiwan. Controlled Drugs Act. Available from: <https://law.moj.gov.tw/ENG/LawClass/LawAll.aspx?pcode=L0030010>
307. Taiwan. Narcotics Hazard Prevention Act. Available from: <https://law.moj.gov.tw/ENG/LawClass/LawAll.aspx?pcode=C0000008>
308. Republic of Tajikistan. Law on Narcotic Drugs, Psychotropic Substances, and Precursors. 1999.
309. Republic of Tajikistan. Criminal Code of the Republic of Tajikistan. 1998.
310. United Republic of Tanzania. Drug Control and Enforcement Act, Cap. 95. 2015. Available from: <https://tanzlii.org/akn/tz/act/2015/5/eng%402019-11-30>
311. United Republic of Tanzania. Drug Control and Enforcement (General) Regulations. 2016. Available from: <https://tanzlii.org/akn/tz/act/gn/2016/173/eng%402016-05-13>
312. Thailand. Narcotics Act B.E. 2522 (1979). Available from: <https://www.thailawforum.com/laws/Narcotics%20Act%202522.pdf>
313. Thailand. Narcotics Control Act B.E. 2519 (1976).
314. Thailand. Psychotropic Substances Act B.E. 2559 (2016). Available from: <https://old.fda.moph.go.th/sites/Narcotics/en/Shared%20Documents/Psychotropic-Substances-Act-B.E.2559.pdf>
315. Thailand. Narcotics Code B.E. 2564 (2021). Available from: <https://en.fda.moph.go.th/media.php?id=517578787425230848&name=NARCOTICS-CODE-ONCB120666.pdf>
316. Togo. Law No. 98-008 on Drug Control. 1998.
317. Kingdom of Tonga. Illicit Drugs Control Act. 2003. Available from: <https://tonga.tradeportal.org/media/IllicitDrugsControlAct_2.pdf>
318. Kingdom of Tonga. Illicit Drugs Control (Amendment) Act. 2012.
319. Kingdom of Tonga. Illicit Drugs Control (Amendment) Act. 2021.
320. Trinidad and Tobago. Dangerous Drugs Act, Chapter 11:25. 1991. Available from: <https://rgd.legalaffairs.gov.tt/Laws2/Alphabetical_List/lawspdfs/11.25.pdf>
321. Trinidad and Tobago. Dangerous Drugs (Amendment) Act. 2000. Available from: <https://www.ttparliament.org/wp-content/uploads/2022/01/a2000-44.pdf>
322. Trinidad and Tobago. Dangerous Drugs (Amendment) Act. 2019. Available from: <https://www.ttparliament.org/wp-content/uploads/2022/01/a2019-24g.pdf>
323. Tunisia. Law No. 92-52 on Narcotics. 1992.
324. Tunisia. Amendment to Law No. 92-52 on Narcotics. 2017.
325. Republic of Turkey. Law No. 2313 on the Control of Narcotic Drugs. 1933.
326. Republic of Turkey. Turkish Penal Code, Law No. 5237. 2004.
327. Republic of Turkey. Law No. 3298 on Narcotic Drugs. 1986.
328. Turkmenistan. Law "On Narcotic Drugs, Psychotropic Substances, Precursors and Preventing Trafficking in Them." 2017.
329. Tuvalu. Dangerous Drugs Act. 1948.
330. Tuvalu. Pharmacy and Therapeutic Products Act. 2016.
331. Uganda. Narcotic Drugs and Psychotropic Substances (Control) Act, 2016. Available from: <https://ulii.org/akn/ug/act/2016/3/eng%402016-02-05>
332. Uganda. Narcotic Drugs and Psychotropic Substances (Control) Act (Commencement) Instrument, 2016. Available from: <https://ulii.org/akn/ug/act/si/2016/14/eng%402016-02-12>
333. Uganda. Narcotic Drugs and Psychotropic Substances (Control) Act, 2024. Available from: <https://ulii.org/akn/ug/act/2024/2/eng%402024-02-23>
334. Ukraine. Law No. 60/95-BP "On Narcotic Drugs, Psychotropic Substances, and Precursors." 1995.
335. Ukraine. Cabinet of Ministers Resolution No. 770 "On Approval of the List of Narcotic Drugs, Psychotropic Substances, and Precursors." 2000. Available from: <https://cmhmda.org.ua/wp-content/uploads/2021/11/list-of-drugs-substances-and-precursors-resolution-cmu-770.pdf>
336. Ukraine. Law "On Measures to Combat Illicit Trafficking in Narcotic Drugs, Psychotropic Substances, and Precursors and Their Abuse."
337. United Arab Emirates. Federal Decree-Law No. 30 of 2021 on Combating Narcotic Drugs and Psychotropic Substances. Available from: <https://uaelegislation.gov.ae/en/legislations/1540>
338. United Arab Emirates. Federal Law No. 14 of 1995 on the Countermeasures against Narcotic Drugs and Psychotropic Substances. Available from: <https://sherloc.unodc.org/cld/uploads/res/document/are/federal-law-no-14-of-1995_html/UAE-fedlaw_14-95.pdf>
339. United States. Controlled Substances Act of 1970. Available from: <https://www.dea.gov/drug-information/csa>
340. United States. Comprehensive Drug Abuse Prevention and Control Act of 1970. Available from: <https://crsreports.congress.gov/product/pdf/r/r45948>
341. DISA Global Solutions. Marijuana Legality by State [Internet]. Houston (TX): DISA Global Solutions; 2024 May 4. Available from: <https://disa.com/marijuana-legality-by-state>
342. Uruguay. Law No. 14.294 on Narcotic Drugs and Psychotropic Substances. 1974.
343. Uruguay. Law No. 17.016 Amending Law No. 14.294. 1998.
344. Uruguay. Law No. 19.172 on the Regulation and Control of Cannabis. 2013.
345. Uzbekistan. Law of the Republic of Uzbekistan "On Narcotic Drugs and Psychotropic Substances." 1999.
346. Uzbekistan. Law No. LRU-971 "On Introducing Amendments and Additions to the Criminal, Criminal Procedure Codes of the Republic of Uzbekistan and the Code of Administrative Responsibility, Aimed at Combating Illicit Trafficking in Narcotic Drugs, Their Analogues or Psychotropic Substances, as well as Potent and Toxic Substances." 2024. Available from: <https://lex.uz/docs/7132353>
347. Yemen. Law No. 3 of 1993 on Combating Illicit Trafficking and Use of Narcotic Drugs and Psychotropic Substances.
348. Zambia. Narcotic Drugs and Psychotropic Substances Act, 2021. Available from: <https://www.parliament.gov.zm/sites/default/files/documents/acts/Act%20No.%2035%20OF%202021%2C%20THE%20NARCOTIC%20DRUGS%20AND%20PSYCHOTROPIC%20SUBSTANCE%20ACT%2C%202021.pdf>
349. Zambia. Cannabis Act, 2021.
350. Zambia. Industrial Hemp Act, 2021.
351. Zimbabwe. Medicines and Allied Substances Control Act [Chapter 15:03]. 1969. Available from: <https://zimlii.org/akn/zw/act/1969/14/eng%402016-12-31>
352. Zimbabwe. Dangerous Drugs Act [Chapter 15:02]. 1955. Available from: <https://zimlii.org/akn/zw/act/1955/28/eng%402016-12-31>
353. Zimbabwe. Criminal Law (Codification and Reform) Act [Chapter 9:23]. 2004.
